# Burden of metabolic dysfunction-associated steatohepatitis, with and without metabolic syndrome, obesity, or diabetes

**DOI:** 10.1186/s12876-026-04787-5

**Published:** 2026-04-06

**Authors:** Elliot B. Tapper, Taylor Ryan, Dave Lewandowski, Jessamine P. Winer-Jones, Machaon Bonafede, Yestle Kim

**Affiliations:** 1 Division of Gastroenterology and Hepatology, University of Michigan, Ann Arbor, MI USA; 2 Real World Evidence, Veradigm, Chicago, IL USA; 3 Health Economics and Outcomes Research, Madrigal Pharmaceuticals, Inc, West Conshohocken, PA USA

**Keywords:** Metabolic dysfunction-associated steatohepatitis, Metabolic syndrome, Nonalcoholic steatohepatitis, Obesity, Type 2 diabetes

## Abstract

**Background:**

Metabolic dysfunction-associated steatohepatitis (MASH) is commonly comorbid with metabolic syndrome; however, MASH can occur in the absence of metabolic syndrome. This retrospective cohort study evaluated the patient characteristics, healthcare utilization, and healthcare costs among patients with MASH with and without metabolic syndrome, obesity, and type 2 diabetes/elevated fasting glucose.

**Methods:**

In a linked dataset of electronic health records (Veradigm Network EHR) and claims (Komodo Health), we identified adults with a MASH diagnosis code (7/1/2018-3/15/2023) and ≥12 months of continuous enrollment pre- and post-MASH diagnosis. Patients with other causes of liver disease, gestational or type 1 diabetes, or bariatric surgery were excluded. Six cohorts were identified: 1) MASH with metabolic syndrome, 2) MASH without metabolic syndrome, 3) MASH with body mass index (BMI) <25, 4) MASH with a BMI <25 and metabolic syndrome, 5) MASH with a BMI <25 without metabolic syndrome, and 6) MASH with a BMI <25 without metabolic syndrome or type 2 diabetes/elevated fasting glucose. We captured demographics, clinical characteristics, all-cause healthcare utilization, and costs.

**Results:**

We identified 98,199 patients with MASH, of which 34.4% did not have metabolic syndrome, and 3.1% had a BMI <25. Mean (standard deviation) annualized all-cause healthcare costs exceeded $19,000 in all cohorts and ranged from $19,018 ($60,359) among patients with a BMI <25 without metabolic syndrome or type 2 diabetes/elevated fasting glucose to $32,592 ($337,462) among patients with metabolic syndrome. Median (interquartile range) costs ranged from $5,336 ($2,085-$14,839) to $11,373 ($4,478-$27,243), and mean costs after excluding the top 1% of spenders ranged from $14,355 ($25,868) to $21,878 ($30,755). Trends were consistent when the analysis was expanded to include patients without a documented BMI.

**Conclusions:**

Metabolic syndrome is commonly comorbid with MASH; however, all-cause healthcare costs remain high even among the subpopulation without metabolic syndrome, elevated BMI, or type 2 diabetes/elevated fasting glucose.

**Supplementary Information:**

The online version contains supplementary material available at 10.1186/s12876-026-04787-5.

## Introduction

It is estimated that 24% of adults in the United States (US) are living with metabolic dysfunction-associated steatotic liver disease (MASLD), and 7%–30% of adults with MASLD have the inflammatory subtype, metabolic dysfunction-associated steatohepatitis (MASH) [1]. MASH, previously known as nonalcoholic steatohepatitis, is a progressive liver disease that can lead to severe complications, such as cirrhosis, hepatocellular cancer, and decompensation requiring liver transplantation [2]. The direct medical costs of MASH in the United States are estimated to be between $24 and $88 billion dollars annually [3, 4]. MASH is also associated with a heavy humanistic burden, with patients reporting a poorer quality of life and more work and activity-related impairments than the general population, and a similar burden to patients with type 2 diabetes [5, 6].

As the liver manifestation of insulin resistance [7, 8], MASH is commonly comorbid with other metabolic conditions. The prevalence of obesity, diabetes, and metabolic syndrome among patients with MASH is estimated to be 82%, 44%, and 71%, respectively [1]. However, metabolic comorbidities are not a prerequisite for MASH, as MASH has been detected in 27% of metabolically healthy non-obese individuals with biopsy-confirmed MASLD [9].

Understanding the costs of MASH is complicated by the presence of these costly metabolic comorbidities. The economic burden of obesity is estimated to be between $170 and $260 billion; the burden of diabetes is estimated to be $300 billion [10–12]. Most prior studies of health burden have attempted to isolate the incremental impact of MASH on diabetes [4, 13–15]. However, the majority of patients with MASH do not have comorbid type 2 diabetes [1]. The objective of this study was to evaluate the clinical characteristics, healthcare resource utilization, and healthcare costs among patients with MASH with and without comorbid metabolic syndrome, obesity, and type 2 diabetes/elevated fasting glucose.

## Methods

### Data sources and ethical compliance

This descriptive, retrospective, observational cohort study leveraged electronic health records (EHR) from the Veradigm Network EHR (Veradigm, Inc., Chicago, IL) linked to insurance claims data from Komodo’s Healthcare Map (Komodo Health, Inc., San Francisco, CA) spanning July 1, 2017, through March 20, 2024. The linked dataset only contains deidentified data as per the de-identification standard defined in Section § 164.514(a) of the Health Insurance Portability and Accountability Act of 1996 (HIPAA) Privacy Rule via a formal determination by a qualified expert [16, 17]. Because this study used only deidentified patient records, it is no longer subject to the HIPAA Privacy Rule and studies using this deidentified dataset are not required to undergo a review by an Institutional Review Board. This study was conducted in compliance with the Declaration of Helsinki and followed the Strengthening the Reporting of Observational Studies in Epidemiology guidelines for reporting observational research studies [18].

### Study cohorts

**Fig. 1:**
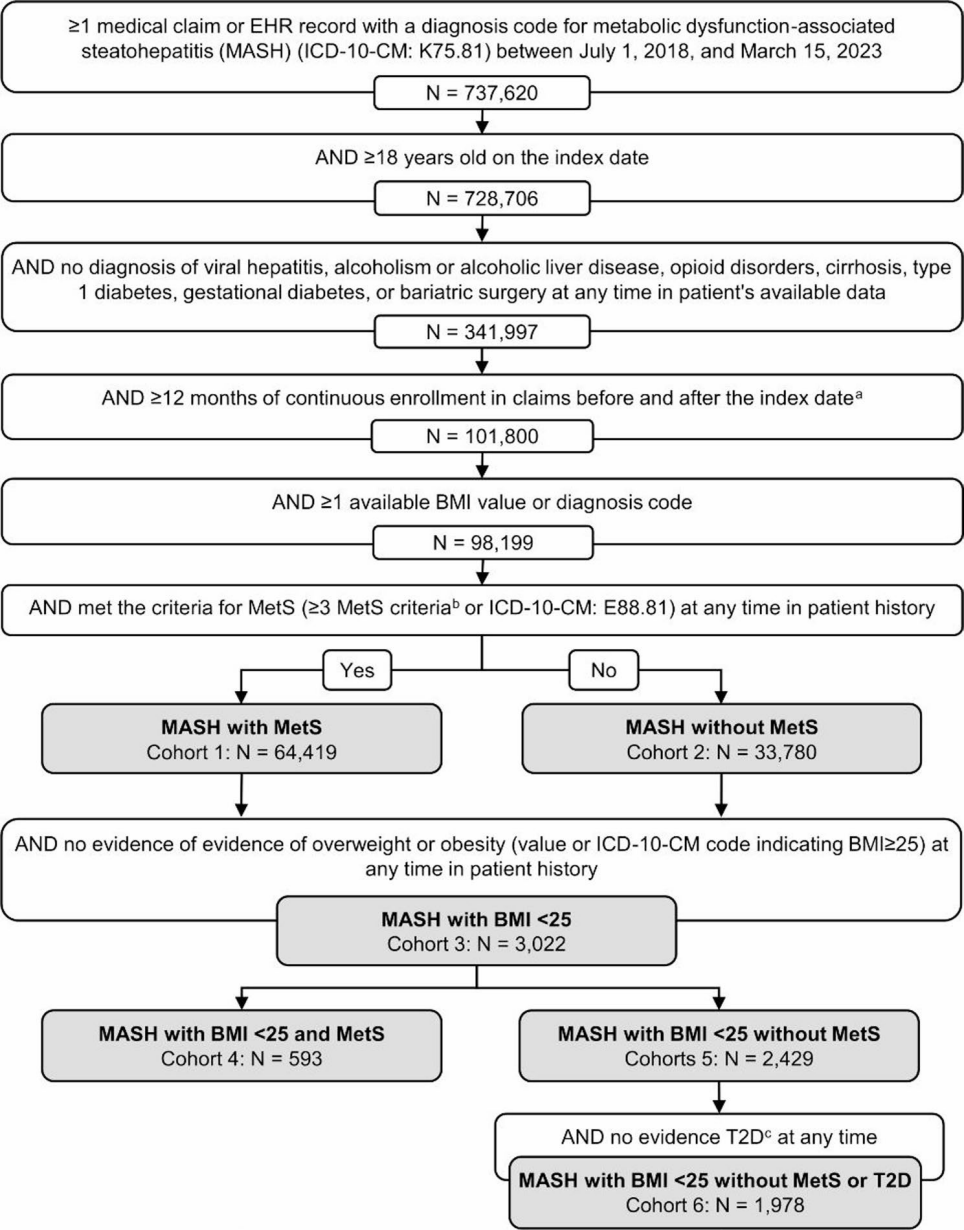
Cohort Identification ICD-10, International Classifications of Disease, Tenth Edition; MetS, metabolic syndrome; T2D, type 2 diabetes (a) The index date was the earliest MASH diagnosis during the patient selection period on which the individual met all study criteria (b) Obesity, high blood pressure, hypercholesterolemia or hyperlipidemia, and T2D/elevated fasting glucose as defined in the methods (c) or elevated fasting glucose

We identified 6 cohorts of patients with MASH: (1) patients with MASH and metabolic syndrome (MASH w/ MetS), (2) patients with MASH without metabolic syndrome (MASH w/o MetS), (3) patients with MASH and body mass index (BMI) < 25 (MASH w/ BMI < 25), (4) patients with MASH, a BMI < 25 and metabolic syndrome (MASH w/ BMI < 25 w/ MetS), (5) patients with MASH and a BMI < 25 and without metabolic syndrome (MASH w/ BMI < 25 w/o MetS), and (6) patients with MASH and a BMI < 25 and without metabolic syndrome or type 2 diabetes/elevated fasting glucose (MASH w/ BMI < 25 w/o MetS w/o T2D). Cohorts 1 and 2 were mutually exclusive and comprised all patients with MASH who met the other study criteria. Cohort 3 was a subset of all patients with MASH. Cohorts 4 and 5 were mutually exclusive subsets of patients in cohort 3. Cohort 6 was a subset of patients in cohort 5. Patient selection criteria and subgroup design are outlined in Fig. 1. A diagram of cohort overlaps are shown in Supplementary Fig. 1.

### Overall patient selection

To identify the study cohorts, we first identified individuals with at least 1 medical claim or EHR record with a diagnosis code for MASH (International Classifications of Diseases, Tenth Edition [ICD-10]: K75.81) and ≥ 1 BMI value or ICD-10 code indicating BMI between July 1, 2018, and March 15, 2023. We excluded individuals who were < 18 years old; with a diagnosis of viral hepatitis, alcoholism or alcoholic liver disease, opioid disorders, cirrhosis, type 1 diabetes, gestational diabetes, or bariatric surgery at any time in their available data. We also required a minimum of 12 months of continuous enrollment in claims before (baseline period) and after (follow-up period) the index date. The codes used in patient selection and cohort assignment are reported in Supplementary Table 1.

### Time periods

The index date was the earliest MASH diagnosis date between July 1, 2018, and March 15, 2023, on which the individual met all the patient selection criteria. The baseline period was the 12 months preceding the index date. The variable length follow-up period began on the index date and ended on the earliest of the following: end of continuous enrollment or March 20, 2024. All patients had at least 12 months of follow-up data. We also used data from the full patient history when identifying certain diagnoses used in patient selection.

### Cohort assignment

We then looked for evidence of metabolic syndrome in all patients. Patients were defined as having metabolic syndrome if, at any time during their patient history, they had a diagnosis of metabolic syndrome (ICD-10: E88.81) or evidence of at least three of the following four conditions: hypertension, hyperlipidemia, obesity, and type 2 diabetes/elevated fasting glucose. Evidence of hypertension included a diagnosis of hypertension or blood pressure of ≥ 130/85. Evidence of hyperlipidemia included a diagnosis of hypercholesterolemia or elevated triglycerides (≥ 150 mg/dL) or reduced high-density lipoprotein [HDL] (< 40 mg/dL for men, < 50 mg/dL for women). Evidence of obesity included any diagnosis of obesity or a record indicating a BMI of ≥ 30. Evidence of type 2 diabetes/elevated fasting glucose included a diagnosis of type 2 diabetes or a fasting blood glucose of ≥ 100 mg/dL. Test thresholds used to define hypertension, hyperlipidemia, and elevated fasting glucose came from the harmonized definition of metabolic syndrome [19]. No priority was given to lab tests or diagnosis codes. When multiple lab tests were recorded on the same day, the mean of the valid values was used. Missing values were not imputed, and medication use was not used to classify patients. Patients were then segmented into two cohorts based on the presence of metabolic syndrome (Fig. 1).

Next, from the initial selection of patients with MASH, we identified a cohort of MASH patients who had no evidence of overweight or obesity either through diagnosis codes or a BMI ≥ 25 at any time during their patient history. This cohort was then segmented into two cohorts by the presence of metabolic syndrome. Finally, within the cohort of patients with MASH who had no evidence of a BMI ≥ 25 and did not meet the criteria for metabolic syndrome, we identified a subset who also did not have a diagnosis of type 2 diabetes or evidence of elevated fasting glucose. Based on clinical input, a diagnosis of prediabetes was not considered sufficient evidence of elevated fasting glucose.

### Study variables

Demographic characteristics, including age, sex, race, ethnicity, geographic region, insurance type, and year of index, were measured on the index date. Clinical characteristics were measured during the 12-month baseline period and included metabolic syndrome-related diagnoses (hypercholesterolemia, hyperlipidemia, primary hypertension, metabolic syndrome, obesity [by diagnosis code or BMI ≥ 30], and type 2 diabetes/elevated fasting glucose), the individual components of the Charlson Comorbidity Index (CCI) [20], other conditions of interest (anemia, anxiety, chronic kidney disease, depression, sleep apnea, and thyroid disease), cancers, and treatments of interest (glucagon-like peptide-1 receptor agonists (GLP-1 RA), antidiabetics [excluding GLP-1 RA], antihypertensives, and statins [high and low/moderate] [21]). Baseline BMI was defined as the value captured in the EHR closest to but preceding the index date during the baseline period. Diagnoses were captured using both EHR and claims data. With the exception of obesity, clinical characteristics were identified solely by diagnosis code (see Supplementary Table 1). Medications were captured using only claims data.

We captured all-cause healthcare utilization and costs in the variable-length follow-up period. Total costs during the follow-up period were captured and then divided by the duration of the follow-up period in years and reported as per-person per-year (PPPY). Due to study requirements, all patients had at least 12 months of follow-up data. Utilization and costs were measured using claims data. We did not calculate proxy costs for encounters that occurred in the EHR but did not have a corresponding claim and we did not adjust for confounding variables or apply any assumptions about cost distributions.

### Exploratory analyses

In an exploratory analysis, we examined the stability of BMI in each of the cohorts. This was achieved by identifying a subset of individuals with ≥ 2 BMI values in the EHR ≥ 6 months apart: 1 in the 6 months preceding the index date, and a second BMI measurement ≥ 6 months after the first BMI measurement. For patients with multiple qualifying BMI records, the baseline measurement was the measurement closest to but preceding the index date, and the follow-up measurement was the measurement closest to but following the date that is 6 months after the baseline measurement. In this subgroup, we identified the number and percentage with at least a 5% increase or decrease in BMI. Diagnosis codes indicating BMI were not used in this portion of the analysis.

### Sensitivity analysis

In the primary analysis, patients were required to have at least one record indicating their BMI; however, this excluded roughly 3.5% of patients who would have otherwise qualified for the study. As a sensitivity analysis, we repeated the study without the requirement for ≥ 1 value or ICD-10 code indicating BMI. Patients without any BMI records were assumed to have a BMI < 25.

### Data analysis

This analysis was descriptive in nature. As the analysis was designed to characterize overlapping subpopulations of interest, study measures were not adjusted for differences in baseline demographics and comparative analyses were not conducted. Categorical variables are reported as n and percent, while continuous variables are reported as means and standard deviation (SD). For cost summary outcomes, we also report medians and interquartile ranges (IQR). Missing data was not imputed. Data analysis was conducted using SAS V9.4.

## Results

This study included 98,199 patients with MASH, among whom 64,419 (65.6%) met the criteria for metabolic syndrome and 33,780 (34.4%) did not meet the criteria for metabolic syndrome (Fig. 1). Of particular interest were the 3,022 (3.1%) individuals with MASH who had evidence of a BMI < 25 and no evidence of a BMI ≥ 25. Among these individuals, 593 (19.6%) had metabolic syndrome, 2,429 (80.4%) did not have metabolic syndrome, and 1,978 (65.5%) did not have metabolic syndrome or type 2 diabetes/elevated fasting glucose. Mean follow-up time was > 2.5 years for all cohorts (Table 1).

### Demographic characteristics

All cohorts were majority female, and the mean age ranged from 48.7 (14.3) years to 62.1 (12.4) years, depending on the cohort (Table 1). When age was assessed categorically, there were shifts in the percentage of the cohorts that were 18–44 years old or 65 + years old (Supplementary Fig. 2). In particular, within the cohort of patients with metabolic syndrome and a BMI < 25, 7.6% were 18–44 years old, and 40.6% were 65 + years old, whereas among all patients with MASH and metabolic syndrome, 19.7% were 18–44 and 19.3% were 65+.

Race data were available for 80.1% (*n* = 78,629) of patients with MASH. Among all patients with MASH, the race breakdown was similar among patients with and without metabolic syndrome, with the majority of patients being White (69.7%–71.5%) (Supplementary Fig. 2). However, the percentage of patients who were White was lower among patients with a BMI < 25 (57.1%), particularly among those with comorbid metabolic syndrome (48.1%). Among all patients with MASH and race data, 6.5% were Asian; however, among patients with a BMI < 25, the percentages of Asian patients ranged from 18.9% among those without metabolic syndrome or type 2 diabetes/elevated fasting glucose to 31.8% among those with metabolic syndrome.

**Table 1:** Demographic Characteristics

	MASH with MetS	MASH without MetS	MASH with BMI < 25	MASH with BMI < 25 with MetS	MASH with BMI < 25 without MetS	MASH with BMI < 25 without MetS or T2D
	*N* = 64,419	*N* = 33,780	*N* = 3,022	*N* = 593	*N* = 2,429	*N* = 1,978
Age, mean (SD)	54.6 (12.7)	48.7 (14.3)	54.7 (15.1)	62.1 (12.4)	52.9 (15.2)	51.6 (15.2)
Female, n (%)	38,561 (59.9)	19,159 (56.7)	2,107 (69.7)	435 (73.4)	1,672 (68.8)	1,360 (68.8)
Race, n (%)						
Asian	3,023 (4.7)	2,100 (6.2)	513 (17.0)	141 (23.8)	372 (15.3)	278 (14.1)
African American/Black	2,911 (4.5)	1,048 (3.1)	69 (2.3)	9 (1.5)	60 (2.5)	45 (2.3)
Other	9,084 (14.1)	4,698 (13.9)	372 (12.3)	80 (13.5)	292 (12.0)	234 (11.8)
White	37,692 (58.5)	18,073 (53.5)	1,269 (42.0)	213 (35.9)	1,056 (43.5)	913 (46.2)
Unknown/Not Reported	11,709 (18.2)	7,861 (23.3)	799 (26.4)	150 (25.3)	649 (26.7)	508 (25.7)
Ethnicity, n (%)						
Hispanic	5,906 (9.2)	2,602 (7.7)	171 (5.7)	40 (6.7)	131 (5.4)	93 (4.7)
Non-Hispanic or Unknown	58,513 (90.8)	31,178 (92.3)	2,851 (94.3)	553 (93.3)	2,298 (94.6)	1,885 (95.3)
Geographic Region, n (%)						
Northeast	12,913 (20.0)	6,966 (20.6)	733 (24.3)	172 (29.0)	561 (23.1)	423 (21.4)
Midwest	11,389 (17.7)	6,268 (18.6)	395 (13.1)	51 (8.6)	344 (14.2)	297 (15.0)
South	26,967 (41.9)	12,977 (38.4)	1,084 (35.9)	207 (34.9)	877 (36.1)	738 (37.3)
West	13,134 (20.4)	7,565 (22.4)	810 (26.8)	163 (27.5)	647 (26.6)	520 (26.3)
Other/Unknown	16 (0.0)	4 (0.0)	0 (0.0)	0 (0.0)	0 (0.0)	0 (0.0)
Insurance Type, n (%)						
Commercial	38,385 (59.6)	22,851 (67.6)	1,781 (58.9)	284 (47.9)	1,497 (61.6)	1,285 (65.0)
Medicare	14,414 (22.4)	4,373 (12.9)	720 (23.8)	222 (37.4)	498 (20.5)	353 (17.8)
Medicaid	11,546 (17.9)	6,498 (19.2)	516 (17.1)	86 (14.5)	430 (17.7)	337 (17.0)
Other/Unknown	74 (0.1)	58 (0.2)	5 (0.2)	1 (0.2)	4 (0.2)	3 (0.2)
Year of Index Date, n (%)						
2018	10,697 (16.6)	4,738 (14.0)	459 (15.2)	110 (18.5)	349 (14.4)	267 (13.5)
2019	14,177 (22.0)	6,969 (20.6)	644 (21.3)	137 (23.1)	507 (20.9)	401 (20.3)
2020	12,711 (19.7)	6,319 (18.7)	546 (18.1)	106 (17.9)	440 (18.1)	357 (18.0)
2021	14,682 (22.8)	8,487 (25.1)	735 (24.3)	119 (20.1)	616 (25.4)	520 (26.3)
2022	11,962 (18.6)	7,145 (21.2)	632 (20.9)	118 (19.9)	514 (21.2)	430 (21.7)
2023	190 (0.3)	122 (0.4)	6 (0.2)	3 (0.5)	3 (0.1)	3 (0.2)
Years of Follow-up, mean (SD)	2.8 (1.3)	2.7 (1.2)	2.7 (1.2)	2.8 (1.3)	2.7 (1.2)	2.6 (1.2)

*MetS* Metabolic syndrome, *SD* Standard deviation, *T2D* Type 2 diabetes

**Table 2:** Baseline Clinical Characteristics

	MASH with MetS	MASH without MetS	MASH with BMI < 25	MASH with BMI < 25 with MetS	MASH with BMI < 25 without MetS	MASH with BMI < 25 without MetS or T2D
	*N* = 64,419	*N* = 33,780	*N* = 3,022	*N* = 593	*N* = 2,429	*N* = 1,978
Baseline BMI, n (%)	26,433 (41.0)	10,077 (29.8)	802 (26.5)	158 (26.6)	644 (26.5)	544 (27.5)
Baseline BMI, mean (SD)	34.0 (4.9)	30.9 (5.8)	21.9 (2.0)	22.2 (1.7)	21.8 (2.0)	21.7 (2.1)
MetS-related conditions, n (%)						
Hypertension (primary)	44,337 (68.8)	11,196 (33.1)	1,044 (34.5)	369 (62.2)	675 (27.8)	493 (24.9)
Hyperlipidemia	41,900 (65.0)	13,653 (40.4)	1,496 (49.5)	427 (72.0)	1,069 (44.0)	773 (39.1)
Obesity	38,705 (60.1)	12,016 (35.6)	0 (0.0)	0 (0.0)	0 (0.0)	0 (0.0)
Diabetes, type 2	31,729 (49.3)	2,145 (6.3)	560 (18.5)	320 (54.0)	240 (9.9)	0 (0.0)
Hypercholesterolemia	13,584 (21.1)	1,954 (5.8)	471 (15.6)	200 (33.7)	271 (11.2)	247 (12.5)
Metabolic syndrome^a^	3,143 (4.9)	0 (0.0)	26 (0.9)	26 (4.4)	0 (0.0)	0 (0.0)
Other Conditions, n (%)						
Thyroid disease	17,962 (27.9)	6,432 (19.0)	735 (24.3)	205 (34.6)	530 (21.8)	410 (20.7)
Anxiety	17,763 (27.6)	8,286 (24.5)	679 (22.5)	116 (19.6)	563 (23.2)	480 (24.3)
Sleep apnea	17,249 (26.8)	4,655 (13.8)	131 (4.3)	31 (5.2)	100 (4.1)	79 (4.0)
Depression	13,150 (20.4)	5,120 (15.2)	366 (12.1)	72 (12.1)	294 (12.1)	245 (12.4)
Chronic kidney disease	5,434 (8.4)	1,048 (3.1)	176 (5.8)	66 (11.1)	110 (4.5)	68 (3.4)
Anemia	4,114 (6.4)	1,518 (4.5)	192 (6.4)	44 (7.4)	148 (6.1)	120 (6.1)
Related Treatments, n (%)						
Any antihypertensive	43,752 (67.9)	11,921 (35.3)	1,035 (34.2)	345 (58.2)	690 (28.4)	508 (25.7)
GLP-1 RA	6,548 (10.2)	401 (1.2)	41 (1.4)	21 (3.5)	20 (0.8)	1 (0.1)
Other antidiabetic	27,179 (42.2)	2,270 (6.7)	391 (12.9)	230 (38.8)	161 (6.6)	11 (0.6)
Statin						
Low/moderate dose^b^	21,126 (32.8)	4,977 (14.7)	697 (23.1)	252 (42.5)	445 (18.3)	315 (15.9)
High dose^b^	10,103 (15.7)	1,851 (5.5)	213 (7.0)	69 (11.6)	144 (5.9)	94 (4.8)

*BMI* Body mass index, *CCI* Charlson Comorbidity Index, *GLP-1 RA* Glucagon-like peptide-1 receptor agonists, *MetS* Metabolic syndrome, *SD* Standard deviation, *T2D* Type 2 diabetes

^a^Defined as patients with an ICD-10-CD diagnosis code of E88.81, ^b^Statin dose intensity was calculated using peak average daily dose for any prescribed statin, with thresholds aligned with Quek, et al. [21]. High doses were defined as follows: Atorvastatin ≥ 30 mg daily; Rosuvastatin ≥ 15 mg daily; Simvastatin ≥ 60 mg daily

### Clinical characteristics

The most prevalent metabolic syndrome-related condition in the baseline period was primary hypertension for patients with MASH and metabolic syndrome, while for all other cohorts, it was hyperlipidemia (Table 2). Among those categorized as MASH with metabolic syndrome, 68.8% had a diagnosis of hypertension, 65.0% had a diagnosis of hyperlipidemia, 60.1% had a diagnosis or BMI indicating obesity, and 49.3% had a diagnosis of type 2 diabetes during the 12 month baseline period. Only 4.9% of patients categorized with metabolic syndrome had a record of the ICD-10 diagnosis code for metabolic syndrome (E88.81).

Conditions and treatments associated with obesity, like sleep apnea, chronic pulmonary disease, and use of GLP-1 RAs, tended to be less common among patients with a BMI < 25 than among all patients with MASH and metabolic syndrome (Table 2 and Supplementary Table 2). The use of treatments related to metabolic syndrome was correlated with the prevalence of metabolic comorbidities. The most prevalent CCI conditions other than those involved in identifying the study cohorts were chronic pulmonary disease, peripheral vascular disease, and any malignancy.

### Weight changes

Roughly 1 in 5 patients with MASH (*n* = 20,893) had 2 BMI measurements at least 6 months apart; however, the majority of patients with sufficient data for analyzing weight changes were patients with MASH and metabolic syndrome (*n* = 16,309). Among the subset of patients who had 2 BMI measurements at least 6 months apart, 64.8%–71.0% had a BMI change of less than 5% (Supplementary Table 3). Among most cohorts, a larger percentage had at least a 5% decrease in BMI compared to a 5% increase in BMI; however, this was the opposite among patients with MASH with a BMI < 25 and without metabolic syndrome and among those without metabolic syndrome or type 2 diabetes/elevated fasting glucose.

### All-cause healthcare utilization and costs

All-cause healthcare utilization during the variable follow-up period was high for all MASH cohorts but tended to be descriptively highest among patients with MASH and metabolic syndrome and lowest among patients with BMI < 25 and without metabolic syndrome or type 2 diabetes/elevated fasting glucose (Supplementary Table 4). In the variable follow-up period, 23.7% of patients with MASH and metabolic syndrome, 16.2% of patients with MASH and a BMI < 25, and 14.3% of patients with MASH, a BMI < 25, and without metabolic syndrome or type 2 diabetes/elevated fasting glucose had at least 1 inpatient admission, while 49.2%, 35.3%, and 34.3% had at least 1 emergency department visit.

**Fig. 2:**
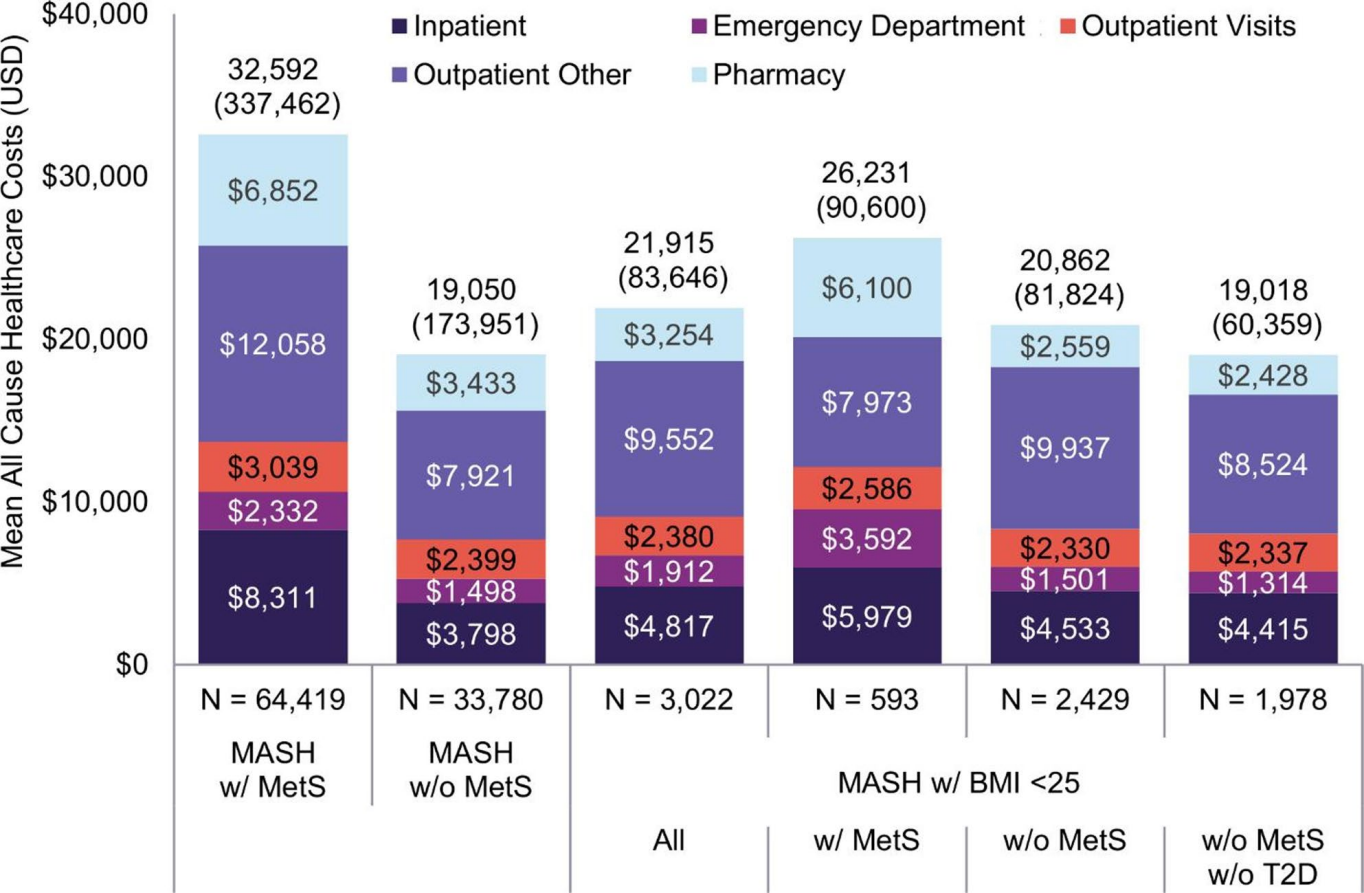
Annualized mean (standard deviation) all-cause healthcare costs in the variable length follow-up period. MetS, metabolic syndrome; T2D, type 2 diabetes; w/, with; w/o, without

Mean (SD) annualized all-cause healthcare costs in the variable follow-up period exceeded $19,000 in all cohorts and ranged from $19,018 ($60,359) PPPY among patients with MASH, a BMI < 25, and without metabolic syndrome or type 2 diabetes/elevated fasting glucose to $32,592 ($337,462) PPPY among patients with MASH and metabolic syndrome (Fig. 2). Median (IQR) costs ranged from $5,336 ($2,085–$14,839) to $11,373 ($4,478–$27,243). Analysis showed that the high standard deviations were driven by a small number of people with very high costs, such as those with hepatocellular carcinoma, decompensated cirrhosis, end-stage liver failure, or high-cost non-liver-related conditions. After removing the top 1% of spenders, mean (SD) costs ranged from $14,355 ($25,868) to $21,878 ($30,755) PPPY, and standard deviations decreased to below $31,000.

Other outpatient services, which included care received outside an office visit, such as laboratory, imaging, and infusion services, made up the largest category of costs for all cohorts, though no specific driver of high costs was identified.

### Sensitivity analysis

In the main analysis, patients were required to have at least 1 record indicating BMI; as a sensitivity analysis, the study was repeated without this requirement. Instead, BMI was handled more similarly to the other metabolic conditions, and patients without a BMI record or diagnosis code were assumed to have a BMI < 25, just as patients without a diagnosis or lab test indicating hypertension were assumed not to have hypertension. The sensitivity analysis included 101,800 patients, and patient selection is outlined in Supplementary Fig. 3.

Overall, patient characteristics and utilization metrics were similar to the main analysis and are reported in Supplementary Fig. 4–5 and Supplementary Tables 5–8. Mean (SD) all-cause costs in the variable follow-up period shifted by less than ±$2,037 for all cohorts except among non-obese MASH patients with metabolic syndrome, for whom mean costs were $26,231 ($90,600) in the main analysis and $38,323 ($472,673) in the sensitivity analysis. A close examination of the cost and utilization tables revealed that this was due to a small number of patients with very high costs categorized as outpatient other. When patients with the top 1% of costs were excluded or when median costs were evaluated, costs in all cohorts shifted by less than ±$1,500 between the main analysis and the sensitivity analysis.

## Discussion

In this real-world analysis of 98,199 patients with MASH, we advance the literature with several key findings. Roughly one-third of patients did not meet the criteria for metabolic syndrome, and around 3% had a BMI < 25. Over 60% of patients with MASH with a BMI < 25 did not have metabolic syndrome or type 2 diabetes/elevated fasting glucose. Weight loss was more common than weight gain in the follow-up period in most cohorts. In the main analysis, mean all-cause healthcare costs exceeded $19,000 for all patient subgroups, and exceeded $14,000 when the top 1% of spenders were excluded. These data suggest that patients with MASH incur high healthcare costs, and while costs are highest among patients with comorbid metabolic syndrome, even non-obese patients without metabolic syndrome have high healthcare utilization and costs.

In this study, mean annualized all-cause healthcare costs for patients with MASH ranged from $19,018 to $32,592, and median costs ranged from $5,336 to $11,373, depending on the burden of metabolic comorbidities. Across the cohorts, inpatient admissions comprised 20%–26% of total costs, while outpatient pharmacy claims comprised 12%–23% of total costs. Other outpatient services made up the largest category of costs, comprising 30%–48% of total costs depending on the cohort. This broad category includes services often associated with diagnosis, monitoring, and management of chronic conditions, such as laboratory tests, imaging services, outpatient procedures, and office-administered medications.

Prior studies have shown that mean all-cause healthcare costs for patients with MASH typically exceed $15,000 per year, and costs are highly dependent on disease state [15, 22, 23]. Mean costs were generally higher among patients with advanced liver disease, such as those with a high Fibrosis-4 score of > 4.12 ($34,667–$54,852) [22, 23] or complex complications like hepatocellular carcinoma ($147,401), decompensated cirrhosis ($181,134), or liver transplant ($300,408) [15]. While these studies all demonstrated a correlation between worsening liver disease and higher costs, they also reported a high prevalence of comorbid metabolic conditions among patients with advanced liver disease, which may be contributing to healthcare costs.

Prior attempts to decouple MASH costs from metabolic comorbidity costs have focused on costs due to type 2 diabetes [4, 13–15]. Two US studies that calculated disease-specific costs from diagnosis codes on administrative claims data estimated that 25%–43% of direct healthcare costs could be attributed to MASH among patients with both MASH and type 2 diabetes [13, 14]. In contrast, a multi-national survey of physician-reported healthcare utilization estimated that 58% (62% in the US subset) of costs could be attributed to MASH [4]. This higher percentage was due to the inclusion of prescription costs for antidiabetics, antihypertensives, and antihyperlipidemics to manage the comorbidities of MASH [4].

Although MASH is widely associated with metabolic syndrome, MASH can develop in the absence of other manifestations of metabolic dysregulation, including obesity [9]. In this study, 34.4% of patients did not meet the criteria for metabolic syndrome at any point in their available records. This suggests that rather than being a downstream complication of metabolic comorbidities, MASH is potentially driven by the same underlying mechanisms, adipose dysfunction and insulin resistance, and may appear before other indicators of metabolic syndrome [7, 8, 24]. While our study was not designed to elucidate mechanisms for MASH among patients with BMI < 25, the demographic profile of these patients (higher percentage female and Asian) tended to align with populations more sensitive to excess visceral fat, which may be present among patients with normal BMI [25–27]. Our analysis suggests that in a small subset of patients, MASH may be the earliest manifestation of metabolic dysfunction. Therefore, it should not be assumed that treatment of comorbid metabolic conditions will fully address the underlying causes of liver fibrosis and result in improved outcomes for patients with MASH.

### Limitations

The primary limitation is that MASH patients were identified by diagnosis code and not by biopsy results. This is because biopsy results are not available in the structured EHR data used in this study, and prior research has shown that liver testing isn’t conducted frequently enough to be captured in a 12-month baseline period [22]. Our use of the K75.81 diagnosis code is consistent with prior literature and recommendations from an expert consensus panel [28–30]. It should also be noted that the data collected for this study came from a period before the multisociety Delphi consensus statement renaming nonalcoholic steatohepatitis to MASH [31]. While the diagnostic criteria and clinical coding did not change [28], the clinical perspective on the disease may have shifted with nomenclature, and some patients categorized as having MASH in this study may not align with the revised clinical perspective.

Another limitation is that, because we are limited by the fields captured in the EHR and claims data sources, we assessed obesity and metabolic disease status using BMI, labs, and diagnosis history rather than waist circumference and clinical assessment [19]. This may result in misidentification of metabolic syndrome and obesity. Specifically, in a 12 month window HbA1c, HDL, and triglyceride results were available for only roughly 10% of the study population. In addition, the use of BMI as a proxy for obesity, along with the use of an anytime window rather than a most recent BMI window, may result in an overestimate of metabolically relevant obesity. However, as all individuals included in this study have a diagnosis of MASH, the potential for misclassification is lower than it would be in a healthy population, as the physical characteristics associated with a discordant BMI and waist circumference (i.e., a high muscle mass) are also protective against the development of MASH [32, 33]. In addition, we looked for evidence of metabolic disease at any point in the patient record rather than looking just in the period preceding the index date. We took this more conservative approach because these metabolic conditions are chronic and are likely present clinically prior to diagnosis. Future studies might take a different approach in patient identification, such as identifying patients without other symptoms of metabolic disease in the year preceding their first MASH diagnosis.

This study is also subject the the standard limitations that apply to all retrospective database studies, such as data entry errors, missing data, and coding limitations. Specifically, in the main analysis, we excluded patients with missing BMI. Our analysis suggests that the missingness in BMI may not be random but instead reflects lower usage of healthcare services compared to patients with available BMI. This may be because these patients are healthier overall and they have less need for healthcare services, or it may reflect barriers to healthcare access. The exclusion of these low utilizing patients might result in overestimating the average cost of MASH. Therefore, in the sensitivity analysis, we retained these patients, and the study results were similar overall. Finally, this study included only individuals with stable insurance coverage. As a result, costs may not be representative of uninsured individuals, those with less stable insurance, and those insured through plans not captured in the data source.

This was a purely descriptive analysis. As the cohorts examined in this study were not mutually exclusive and were anticipated to represent demographically and clinically distinct subpopulations, we did not conduct any matching, multivariable adjustment, or statistical analysis. Because of this, readers should be cautious when comparing between cohorts and interpreting the results. Differences in costs should not be attributed specifically to the clinical comorbidities used to segment the subgroups.

## Conclusions

Metabolic syndrome is commonly comorbid with MASH; however, from this descriptive analysis, roughly one-third of patients with MASH do not meet the criteria for metabolic syndrome. Annualized all-cause costs among patients with MASH remain high even among the subpopulation without elevated BMI, type 2 diabetes/elevated fasting glucose, or metabolic syndrome.

## Supplementary Information

Supplementary Material 1.

## Data Availability

The data that support the findings of this study are available from Veradigm and Komodo Health but restrictions apply to the availability of these data, which were used under license for the current study, and so are not publicly available. Data are however available from the authors upon reasonable request and with permission of Veradigm and Komodo Health.

## Abbreviations

BMI: Body mass index
CCI: Charlson Comorbidity Index
EHR: Electronic health records
GLP-1 RA: Glucagon-like peptide-1 receptor agonists
HDL: High-density lipoprotein
HIPAA: Health Insurance Portability and Accountability Act of 1996
ICD-10: International Classification of Diseases, Tenth Edition
IQR: Interquartile ranges
MASH: Metabolic dysfunction-associated steatohepatitis
MASLD: Metabolic dysfunction-associated steatotic liver disease
PPPY: Per-person per-year
SD: Standard deviation
US: United States
w/ BMI <25: with BMI <25
w/ MetS: With metabolic syndrome
w/o MetS: Without metabolic syndrome
w/o T2D: without type 2 diabetes
