# Supplementary material for: Burden of metabolic dysfunction-associated steatohepatitis, with and without metabolic syndrome, obesity, or diabetes
Source: BMC Gastroenterol. 2026 Apr 6;26:301. doi: 10.1186/s12876-026-04787-5 (PMC13182114; doi:10.1186/s12876-026-04787-5)
Supplement: Supplementary file 1 — Supplementary Material 1. [file 12876_2026_4787_MOESM1_ESM.pdf]

Supplementary Materials for: *Burden of Metabolic Dysfunction-Associated Steatohepatitis, with and without Metabolic Syndrome, Obesity, or Diabetes*

Authors: Elliot B. Tapper, Taylor Ryan, Dave Lewandowski, Jessamine P. Winer-Jones, Machaon Bonafede, Yestle Kim

Listed in order they are referenced in the main text:

Supplementary Table 1. Codes for study execution

Supplementary Figure 1. Patient Cohorts

Supplementary Figure 2. Distribution of (A) Age and (B) Race[a]

Supplementary Table 2. Additional Baseline Clinical Characteristics

Supplementary Table 3. Changes in Body Mass Index (BMI) Among Patients with 2 BMI Measurements ≥6 Months Apart.

Supplementary Table 4. All-Cause Healthcare Resource Utilization in the Variable Follow-Up Period Reported as Per-Person Per-Year

Sensitivity Analysis:

Supplementary Figure 3. Cohort Identification: Sensitivity Analysis

Supplementary Table 5. Demographic Characteristics – Sensitivity Analysis

Supplementary Figure 4. Distribution of (A) Age and (B) Racea – Sensitivity Analysis.

Supplementary Table 6. Baseline Clinical Characteristics – Sensitivity Analysis

Supplementary Table 7. Additional Baseline Clinical Characteristics – Sensitivity Analysis

Supplementary Figure 5. Annualized Mean (Standard Deviation) All-cause Healthcare Costs in the Variable Length Follow-up Period – Sensitivity Analysis.

Supplementary Table 8. All-Cause Healthcare Resource Utilization in the Variable Follow-Up Period Reported as Per-Person Per-Year – Sensitivity Analysis

*Supplementary Table 1. Codes for study execution. Note: For the code lists below, x is used as a wildcard for ICD-9 codes and * is used as a wildcard for ICD-10 codes*

Study Section	Description	Code Type	Code
Inclusion criteria	MASH[a]	ICD-10-CM	K75.81
Exclusion criteria	Alcoholism[b]	ICD-9-CM	265.2, 291.x (except 291.0), 303.x, 305.0x, 357.5, 425.5, 535.3x, 571.0, 571.1, 571.3, 980.x, V11.3
		ICD-10-CM	E52, F10*, G62.1, I42.6, K29.2*, T51*, Z50.2, Z71.4*, Z72.1
	Alcoholic liver disease	ICD-9-CM	571.1, 571.3
		ICD-10-CM	K70*
	Bariatric Surgery	CPT	43644, 43645, 43770, 43771, 43773, 43775, 43842, 43843, 43845, 43846, 43847, 43848, 43886, 43887, 43888
		ICD-10-PCS	0D1607A, 0D160JA, 0D160KA, 0D160ZA, 0D164Z9, 0D164ZA, 0D164ZB, 0D190ZB, 0DB60ZZ, 0DB64Z3, 0DB64ZZ, 0DP64CZ, 0DP64JZ, 0DV60CZ, 0DV60DZ, 0DV60ZZ, 0DV64CZ, 0DV64ZZ, 0DW60DZ, 0DW60JZ, 0DW64CZ
		ICD-9-CM	539.x
		ICD-10-CM	K95*
	Cirrhosis	ICD-9-CM	5712, 5713
		ICD-10-CM	K70.3*, K17, K74.6*
	Gestational diabetes	ICD-9-CM	648.0x, V12.21
		ICD-10-CM	O24.4*, Z86.32
	Opioid disorders	ICD-9-CM	304.0x, 304.7x, 305.5x
		ICD-10-CM	F11*
	Type 1 diabetes	ICD-9-CM	250.1, 250.03, 250.11, 250.13, 250.21, 250.23, 250.31, 250.33, 250.41, 250.43, 250.51, 250.53, 250.61, 250.63, 250.71, 250.73, 250.81, 250.83, 250.91, 250.93
		ICD-10-CM	E10*, O24.0*
	Viral hepatitis	ICD-9-CM	070.x, 573.1
		ICD-10-CM	B15*, B16*, B17*, B18*, B19*, B94.2, O98.4*
Cohort stratification: Diagnoses	Hypercholesteremia or Hyperlipidemia	ICD-10-CM	E78.0*, E78.1, E78.2, E78.3, E78.41, E78.49, E78.5, E78.6
	Hypertension	ICD-10-CM	I10*, I11*, I12*, I13*, I15*
	Metabolic Syndrome	ICD-10-CM	E88.81
	Obesity	ICD-10-CM	E66*, Z68.3*, Z68.4*
	Overweight	ICD-10-CM	Z68.2*
	Type 2 diabetes	ICD-10-CM	E11*
Cohort stratification: labs	Triglycerides	CPT	84478
		LOINC	12951-0, 14927-8, 1644-4, 17081-1, 2571-8, 3043-7, 3048-6, 3049-4, 30524-3, 35217-9, 47210-0, 70218-3, 94865-3, 96598-8

Study Section	Description	Code Type	Code
	High-density lipoprotein	CPT	83718
		LOINC	14646-4, 14813-0, 18263-4, 2085-9, 2086-7, 2573-4, 35197-3, 49130-8, 49748-7, 96596-2
	Fasting blood glucose	LOINC	101476-0, 10450-5, 14770-2, 14771-0, 1554-5, 1556-0, 1557-8, 1558-6, 17865-7, 35184-1, 41604-0, 76629-5, 77145-1
Clinical Characteristics: Metabolic Syndrom-related Conditions	Diabetes, type 2	ICD-9-CM	250.00, 250.02, 250.10, 250.12, 250.20, 250.22, 250.30, 250.32, 250.40, 250.42, 250.50, 250.52, 250.60, 250.62, 250.70, 250.72, 250.80, 250.82, 250.90, 250.92
		ICD-10-CM	E11*, O24.1*
	Hypercholesterolemia	ICD-9-CM	272.0
		ICD-10-CM	E78.0*
	Hyperlipidemia	ICD-9-CM	272.1, 272.2, 272.3, 272.4, 272.5
		ICD-10-CM	E78.1, E78.2, E78.3, E78.41, E78.49, E78.5, E78.6
	Hypertension (primary)	ICD-9-CM	401.x
		ICD-10-CM	I10
	Metabolic syndrome	ICD-9-CM	277.7
		ICD-10-CM	E88.81
	Obesity	ICD-9-CM	278.x, V85.3x, V85.4x
		ICD-10-CM	E66*, Z68.3*, Z68.4*
Clinical Characteristics: Other Conditions	Anemia	ICD-9-CM	280.x
		ICD-10-CM	D50*
	Anxiety	ICD-9-CM	300.0x, 300.2x, 300.3, 300.4, 300.5, 300.6, 300.7
		ICD-10-CM	F40*, F41*, F42*, F43*
	Chronic kidney disease	ICD-9-CM	403.x, 404.x, 585.x
		ICD-10-CM	I12*, I13*, N18*
	Depression	ICD-9-CM	296.20, 296.21, 296.22, 296.23, 296.24, 296.25, 296.30, 296.31, 296.32, 296.33, 296.34, 296.35
		ICD-10-CM	F32*, F33*, F34.1
	Sleep apnea	ICD-9-CM	327.2x
		ICD-10-CM	G47.3*
	Thyroid disease	ICD-9-CM	240.x, 241.x, 242.x, 243.x, 245.x, 246.x
		ICD-10-CM	E00*, E01*, E02, E03*, E04*, E05*, E06*, E07*

Study Section	Description	Code Type	Code
Clinical Characteristics: Related Treatments	Antihypertensives	Generic name	acebutolol hcl, aliskiren hemifumarate, aliskiren/valsartan, amiloride hcl/hydrochlorothiazide, amlodipine benzoate, amlodipine besylate, atenolol, azilsartan medoxomil, benazepril hcl, bendroflumethiazide, betaxolol hcl, bisoprolol fumarate, bumetanide, candesartan cilexetil, captopril, carteolol hcl, carvedilol, chlorothiazide, chlorthalidone, clonidine, diltiazem hcl, diltiazem malate, doxazosin mesylate, enalapril maleate, eplerenone, eprosartan mesylate, ethacrynic acid, felodipine, finerenone, fosinopril sodium, furosemide, guanabenz acetate, guanadrel sulfate, guanethidine sulfate, guanfacine hcl, hydralazine hcl, hydrochlorothiazide, hydroflumethiazide, indapamide, irbesartan, isradipine, labetalol hcl, levamlodipine maleate, lisinopril, losartan potassium, mecamylamine hcl, methyclothiazide, methyldopa, metolazone, metoprolol succinate, metoprolol tartrate, minoxidil, moexipril hcl, nadolol, nebivolol hcl, nicardipine hcl, nifedipine, nisoldipine, nitroprusside sodium, olmesartan medoxomil, penbutolol sulfate, perindopril erbumine, phenoxybenzamine hcl, pindolol, polythiazide, prazosin hcl, propranolol hcl, quinapril hcl, ramipril, reserpine, sotalol hcl, spironolactone, telmisartan, terazosin hcl, timolol maleate, torsemide, trandolapril, triamterene/hydrochlorothiazide, trichlormethiazide, valsartan, verapamil hcl
	Glucagon-like peptide-1 receptor agonists	Generic name	tirzepatide, dulaglutide, exenatide, liraglutide, lixisenatide, semaglutide, albiglutide
	Other antidiabetics	Generic name	acarbose, acetohexamide, alogliptin benzoate, bromocriptine mesylate, canagliflozin, chlorpropamide, dapagliflozin propanediol, empagliflozin, glimepiride, glipizide, glyburide, insulin , linagliptin, metformin hcl, mifepristone, miglitol, nateglinide, pioglitazone hcl, pramlintide acetate, repaglinide, rosiglitazone maleate, saxagliptin hcl, sitagliptin phosphate, sotagliflozin, tolazamide, tolbutamide, troglitazone
	Statins	Generic name	atorvastatin , cerivastatin , fluvastatin , lovastatin, pitavastatin , pravastatin , rosuvastatin, simvastatin
Clinical Characteristics: Liver-Related Conditions	Autoimmune hepatitis	ICD-9-CM	571.42
		ICD-10-CM	K75.4
	Gastroesophageal varices	ICD-9-CM	456.0, 456.1, 456.8, 456.20, 456.21
		ICD-10-CM	I85*
	Hepatic encephalopathy	ICD-9-CM	572.2
		ICD-10-CM	K71.11, K72.01, K72.11, K72.90, K72.91
	Ascites	ICD-9-CM	789.5x
		ICD-10-CM	R18*, K15.1
Clinical Characteristics: Cancers	Bladder cancer	ICD-9-CM	188.x
		ICD-10-CM	C67*
	Breast cancer	ICD-9-CM	174.x, 175.x
		ICD-10-CM	C50*
	Colorectal cancer	ICD-9-CM	153.x, 154.x
		ICD-10-CM	C18*, C19, C20, C21*

Study Section	Description	Code Type	Code
	Kidney cancer	ICD-9-CM	189.x
		ICD-10-CM	C64*
	Liver cancer	ICD-9-CM	155.x
		ICD-10-CM	C22*
	Ovarian cancer	ICD-9-CM	183.x
		ICD-10-CM	C56*
	Pancreatic cancer	ICD-9-CM	157.x
		ICD-10-CM	C25*
	Prostate cancer	ICD-9-CM	185
		ICD-10-CM	C61
	Stomach cancer	ICD-9-CM	151.x
		ICD-10-CM	C16*
	Uterine cancer	ICD-9-CM	182.x
		ICD-10-CM	C54*

CPT, Current Procedural Terminology; ICD-9-CM, International Classification of Diseases, 9th Edition, Clinical Modification; ICD-10-CM, International Classification of Diseases, 10th Edition, Clinical Modification; ICD-10-PCS, International Classification of Diseases, 10th Edition, Procedure Coding System; MASH, metabolic dysfunction-associated steatohepatitis; NDC, National Drug Codes

a) Previously known as nonalcoholic steatohepatitis (NASH)

b) The presence of a code for niacin deficiency was taken as evidence of alcohol use disorder

Supplementary Figure 1. Patient Cohorts

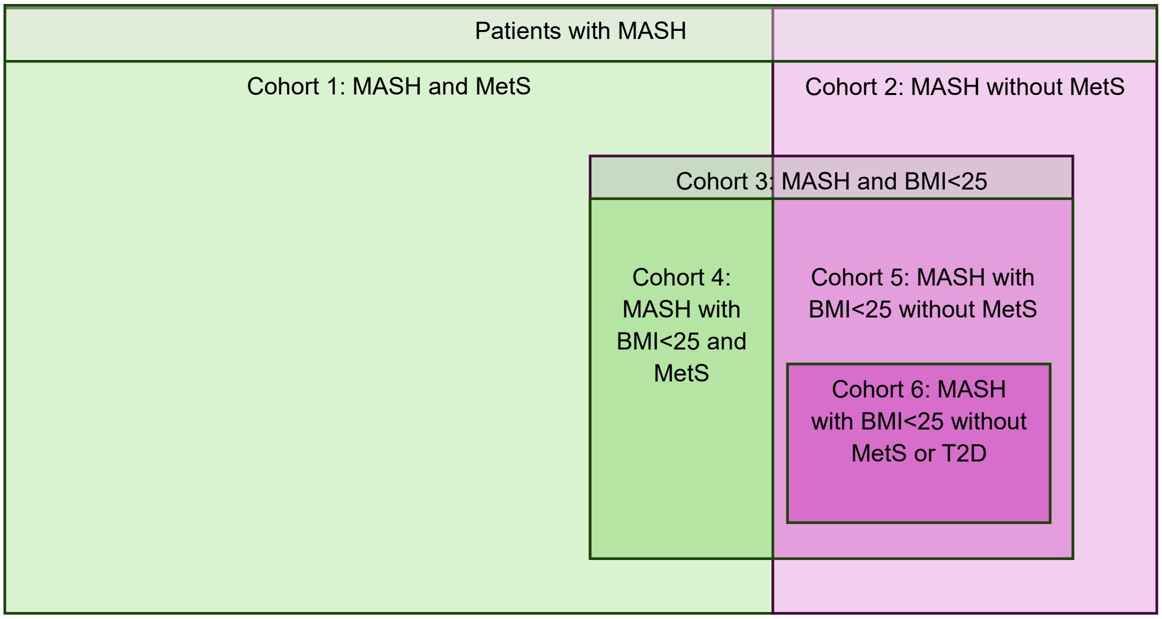

MetS, metabolic syndrome; T2D, type 2 diabetes

Supplementary Figure 2. Distribution of (A) Age and (B) Race[a]

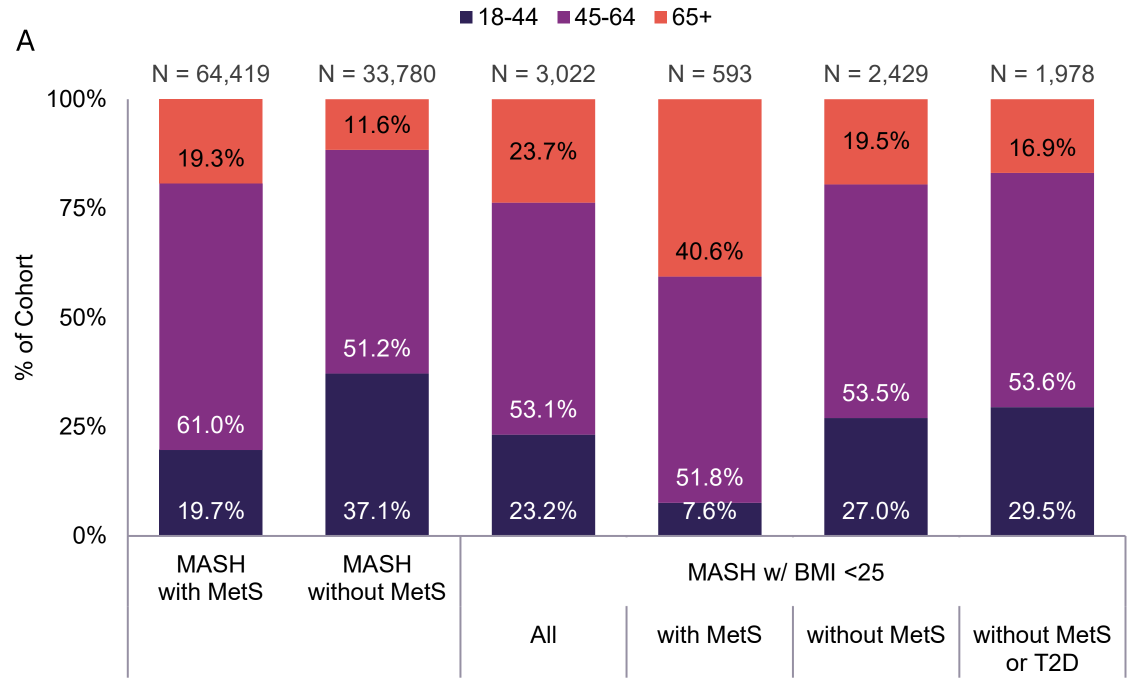

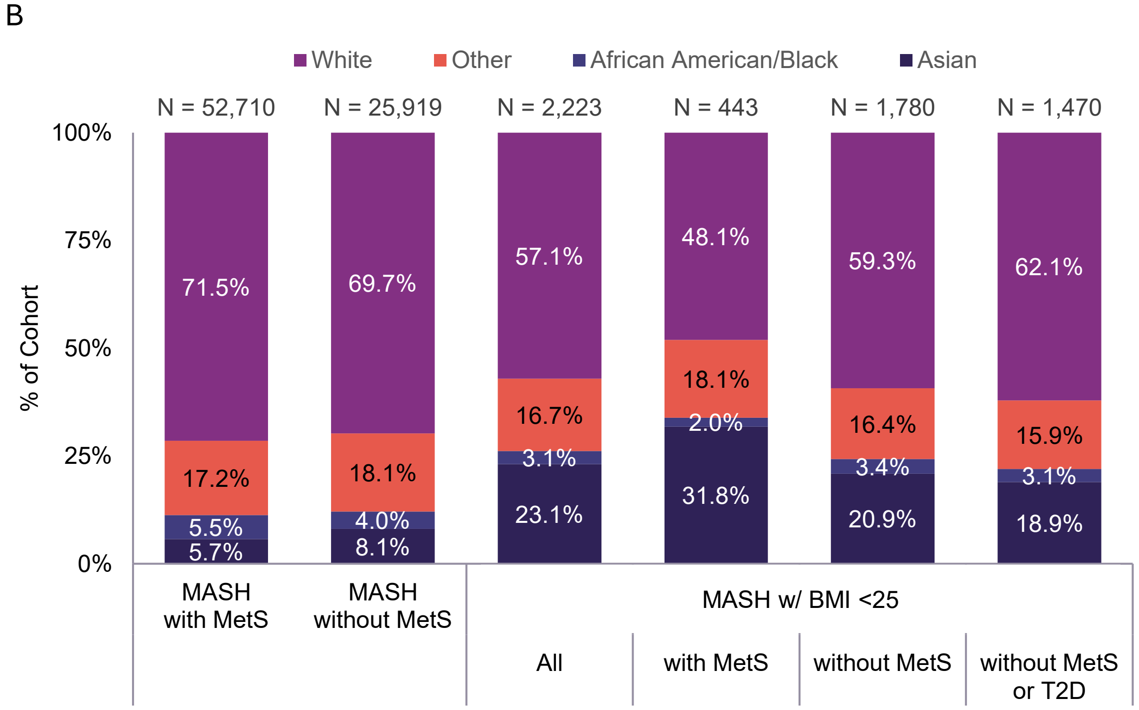

[a]Race was missing in 19,570 individuals and percentages are calculated only among those with available race data. MetS, metabolic syndrome; T2D, type 2 diabetes

Supplementary Table 2. Additional Baseline Clinical Characteristics

	MASH with MetS	MASH without MetS	MASH with BMI <25	MASH with BMI <25 with MetS	MASH with BMI <25 without MetS	MASH with BMI <25 without MetS or T2D
	N = 64,419	N = 33,780	N = 3,022	N = 593	N = 2,429	N = 1,978
CCI Conditions, n (%)						
Mild liver disease[a]	31,618 (49.1)	15,174 (44.9)	1,218 (40.3)	253 (42.7)	965 (39.7)	804 (40.6)
Diabetes without chronic complication[a]	31,039 (48.2)	2,095 (6.2)	543 (18.0)	310 (52.3)	233 (9.6)	0 (0.0)
Chronic pulmonary disease	13,600 (21.1)	4,878 (14.4)	425 (14.1)	100 (16.9)	325 (13.4)	262 (13.2)
Diabetes with chronic complication[a]	10,400 (16.1)	493 (1.5)	166 (5.5)	101 (17.0)	65 (2.7)	1 (0.1)
Peripheral vascular disease	5,925 (9.2)	1,386 (4.1)	256 (8.5)	91 (15.3)	165 (6.8)	109 (5.5)
Renal disease[a]	5,278 (8.2)	1,001 (3.0)	170 (5.6)	62 (10.5)	108 (4.4)	66 (3.3)
Any malignancy (Incl. blood, excl. skin)	4,752 (7.4)	1,738 (5.1)	229 (7.6)	51 (8.6)	178 (7.3)	136 (6.9)
Cerebrovascular disease	3,800 (5.9)	992 (2.9)	174 (5.8)	52 (8.8)	122 (5.0)	88 (4.4)
Rheumatic disease	3,633 (5.6)	1,587 (4.7)	149 (4.9)	32 (5.4)	117 (4.8)	91 (4.6)
Congestive heart failure[a]	3,436 (5.3)	630 (1.9)	91 (3.0)	31 (5.2)	60 (2.5)	44 (2.2)
Myocardial infarction	1,643 (2.6)	278 (0.8)	48 (1.6)	14 (2.4)	34 (1.4)	22 (1.1)
Peptic ulcer disease	1,186 (1.8)	457 (1.4)	61 (2.0)	15 (2.5)	46 (1.9)	39 (2.0)
Metastatic solid tumor	657 (1.0)	324 (1.0)	38 (1.3)	9 (1.5)	29 (1.2)	22 (1.1)
Dementia	477 (0.7)	121 (0.4)	34 (1.1)	12 (2.0)	22 (0.9)	14 (0.7)
Hemiplegia or paraplegia	403 (0.6)	162 (0.5)	34 (1.1)	5 (0.8)	29 (1.2)	24 (1.2)
HIV/AIDS	269 (0.4)	146 (0.4)	14 (0.5)	2 (0.3)	12 (0.5)	11 (0.6)
Moderate or severe liver disease[a]	267 (0.4)	103 (0.3)	12 (0.4)	1 (0.2)	11 (0.5)	8 (0.4)

	MASH with MetS	MASH without MetS	MASH with BMI <25	MASH with BMI <25 with MetS	MASH with BMI <25 without MetS	MASH with BMI <25 without MetS or T2D
	N = 64,419	N = 33,780	N = 3,022	N = 593	N = 2,429	N = 1,978
Liver-Related Conditions (N, %)						
Autoimmune hepatitis	301 (0.5)	189 (0.6)	13 (0.4)	1 (0.2)	12 (0.5)	10 (0.5)
Gastroesophageal varices	96 (0.1)	39 (0.1)	2 (0.1)	0 (0.0)	2 (0.1)	1 (0.1)
Hepatic encephalopathy	45 (0.1)	20 (0.1)	4 (0.1)	1 (0.2)	3 (0.1)	2 (0.1)
Ascites	274 (0.4)	134 (0.4)	18 (0.6)	1 (0.2)	17 (0.7)	14 (0.7)
Cancers, n (%)						
Breast cancer	1,627 (2.5)	652 (1.9)	99 (3.3)	24 (4.0)	75 (3.1)	55 (2.8)
Prostate cancer	656 (1.0)	211 (0.6)	17 (0.6)	5 (0.8)	12 (0.5)	9 (0.5)
Colorectal cancer	403 (0.6)	181 (0.5)	25 (0.8)	8 (1.3)	17 (0.7)	13 (0.7)
Kidney cancer	299 (0.5)	77 (0.2)	4 (0.1)	0 (0.0)	4 (0.2)	4 (0.2)
Uterine cancer	283 (0.4)	86 (0.3)	13 (0.4)	1 (0.2)	12 (0.5)	12 (0.6)
Bladder cancer	197 (0.3)	43 (0.1)	5 (0.2)	1 (0.2)	4 (0.2)	2 (0.1)
Ovarian cancer	119 (0.2)	60 (0.2)	7 (0.2)	1 (0.2)	6 (0.2)	6 (0.3)
Liver cancer	89 (0.1)	23 (0.1)	2 (0.1)	0 (0.0)	2 (0.1)	1 (0.1)
Hepatocellular carcinoma	36 (0.1)	11 (0.0)	1 (0.0)	0 (0.0)	1 (0.0)	0 (0.0)
Pancreatic cancer	76 (0.1)	37 (0.1)	9 (0.3)	1 (0.2)	8 (0.3)	6 (0.3)
Stomach cancer	34 (0.1)	10 (0.0)	1 (0.0)	0 (0.0)	1 (0.0)	1 (0.1)

MetS, metabolic syndrome; SD, standard deviation; T2D, type 2 diabetes a) The code sets to define these conditions overlap with those used to define the study cohorts.

Supplementary Table 3. Changes in Body Mass Index (BMI) Among Patients with 2 BMI Measurements ≥6 Months Apart.

	MASH with MetS	MASH without MetS	MASH with BMI <25	MASH with BMI <25 with MetS	MASH with BMI <25 without MetS	MASH with BMI <25 without MetS or T2D
	N = 64,419	N = 33,780	N = 3,022	N = 593	N = 2,429	N = 1,978
2 BMIs ≥6 months apart[a], n (%)	16,309 (25.3)	4,584 (13.6)	361 (11.9)	81 (13.7)	280 (11.5)	241 (12.2)
<5% change in BMI, n (%)	10,561 (64.8)	2,978 (65.0)	252 (69.8)	55 (67.9)	197 (70.4)	171 (71.0)
≥5% decrease in BMI, n (%)	3,684 (22.6)	889 (19.4)	58 (16.1)	20 (24.7)	38 (13.6)	31 (12.9)
≥5% increase in BMI, n (%)	2,064 (12.7)	717 (15.6)	51 (14.1)	6 (7.4)	45 (16.1)	39 (16.2)

MetS, metabolic syndrome; T2D, type 2 diabetes [a] First BMI measurement must be in the 6 months preceding the index date, and the second BMI measurement must be ≥6 months after the first BMI measurement.

Supplementary Table 4. All-Cause Healthcare Resource Utilization in the Variable Follow-Up Period Reported as Per-Person Per-Year

	MASH with MetS	Mash without MetS	MASH with BMI <25	MASH with BMI <25 with MetS	MASH with BMI <25 without MetS	MASH with BMI <25 without MetS or T2D
	N = 64,419	N = 33,780	N = 3,022	N = 593	N = 2,429	N = 1,978
Inpatient admission						
Had an admission, n (%)	15,253 (23.7)	5,108 (15.1)	489 (16.2)	115 (19.4)	374 (15.4)	282 (14.3)
Number of admissions, mean (SD)	0.3 (1.2)	0.1 (0.9)	0.3 (2.0)	0.5 (3.3)	0.2 (1.6)	0.2 (1.4)
Length of stay per hospitalized patient, mean (SD)	7.9 (21.9)	7.1 (20.3)	10.0 (35.5)	13.3 (38.6)	9.0 (34.5)	6.8 (8.6)
Costs, mean (SD) among all patients	$8,311 ($207,965)	$3,798 ($50,258)	$4,817 ($28,889)	$5,979 ($28,893)	$4,533 ($28,881)	$4,415 ($29,844)
Costs, median among all patients	$0	$0	$0	$0	$0	$0
Costs, mean (SD) for patients with an admission	$34,491 ($422,600)	$24,404 ($125,411)	$28,826 ($65,590)	$29,548 ($58,558)	$28,601 ($67,632)	$29,906 ($72,601)
Costs, median for patients with an admission	$7,772	$6,600	$7,995	$8,735	$7,692	$7,163
Emergency Department (ED)						
Had an ED visit, n (%)	31,704 (49.2)	13,844 (41.0)	1,066 (35.3)	217 (36.6)	849 (35.0)	679 (34.3)
Number of ED visits, mean (SD) among all patients	0.6 (1.5)	0.4 (1.0)	0.4 (0.8)	0.3 (0.7)	0.4 (0.8)	0.4 (0.8)
Costs, mean (SD) among all patients	$2,332 ($13,926)	$1,498 ($7,208)	$1,912 ($20,975)	$3,592 ($41,529)	$1,501 ($11,200)	$1,314 ($4,726)
Costs, median among all patients	$0	$0	$0	$0	$0	$0
Costs, mean (SD) for patients with an ED visit	$4,745 ($19,572)	$3,662 ($10,912)	$5,429 ($35,077)	$9,816 ($68,205)	$4,306 ($18,645)	$3,839 ($7,454)
Costs, median for patients with an ED visit	$1,461	$1,231	$1,184	$1,397	$1,153	$1,169

	MASH with MetS	Mash without MetS	MASH with BMI <25	MASH with BMI <25 with MetS	MASH with BMI <25 without MetS	MASH with BMI <25 without MetS or T2D
	N = 64,419	N = 33,780	N = 3,022	N = 593	N = 2,429	N = 1,978
Outpatient (OP) Visits						
Had an OP visit, n (%)	64,286 (99.8)	33,687 (99.7)	3,012 (99.7)	590 (99.5)	2,422 (99.7)	1,973 (99.7)
Number of OP visits, mean (SD)	16.8 (13.6)	13.1 (11.9)	13.8 (12.2)	15.1 (13.1)	13.5 (11.9)	13.3 (11.9)
Costs, mean (SD) among all patients	$3,039 ($8,440)	$2,399 ($7,174)	$2,380 ($4,818)	$2,586 ($4,146)	$2,330 ($4,966)	$2,337 ($5,236)
Costs, median among all patients	$1,452	$1,028	$1,136	$1,316	$1,104	$1,077
OP Other						
Had an OP other service, n (%)	64,212 (99.7)	33,525 (99.2)	3,002 (99.3)	592 (99.8)	2,410 (99.2)	1,961 (99.1)
Number of OP other services, mean (SD)	15.6 (26.7)	10.4 (17.3)	12.9 (26.2)	17.5 (37.8)	11.8 (22.4)	11.2 (21.4)
Costs, mean (SD) among all patients	$12,058 ($75,703)	$7,921 ($41,197)	$9,552 ($63,351)	$7,973 ($18,853)	$9,937 ($70,040)	$8,524 ($45,659)
Costs, median among all patients	$2,951	$1,827	$1,932	$2,350	$1,812	$1,754
Pharmacy						
Had a pharmacy claim, n (%)	63,753 (99.0)	33,079 (97.9)	2,942 (97.4)	584 (98.5)	2,358 (97.1)	1,917 (96.9)
Number of unique NDCs, mean (SD)	13.2 (9.1)	8.5 (6.7)	8.6 (6.8)	10.7 (7.2)	8.1 (6.6)	7.6 (6.2)
Costs, mean (SD) among all patients	$6,852 ($250,069)	$3,433 ($159,012)	$3,254 ($32,272)	$6,100 ($69,843)	$2,559 ($10,117)	$2,428 ($10,484)
Costs, median among all patients	$1,233	$306	$331	$599	$290	$242

	MASH with MetS	Mash without MetS	MASH with BMI <25	MASH with BMI <25 with MetS	MASH with BMI <25 without MetS	MASH with BMI <25 without MetS or T2D
	N = 64,419	N = 33,780	N = 3,022	N = 593	N = 2,429	N = 1,978
Total Costs Among All Patients						
Total Costs, mean (SD)	$32,592 ($337,462)	$19,050 ($173,951)	$21,915 ($83,646)	$26,231 ($90,600)	$20,862 ($81,824)	$19,018 ($60,359)
Total Costs, median (IQR)	$11,373 ($4,478-$27,243)	$5,810 ($2,183-$15,814)	$6,025 ($2,369-$16,298)	$8,064 ($3,320-$20,098)	$5,692 ($2,226-$15,431)	$5,336 ($2,085-$14,839)
Total Costs After Removing Top 1% of Spenders						
Total Costs, mean (SD)	$21,878 ($30,755)	$14,615 ($25,304)	$15,265 ($26,596)	$17,678 ($27,967)	$14,681 ($26,219)	$14,355 ($25,868)
Total Costs, median (IQR)	$11,041 ($4,377-$25,913)	$5,702 ($2,152-$15,346)	$5,902 ($2,336-$15,506)	$7,860 ($3,282-$19,262)	$5,559 ($2,196-$14,778)	$5,170 ($2,069-$14,357)

Supplementary Figure 3. Cohort Identification: Sensitivity Analysis

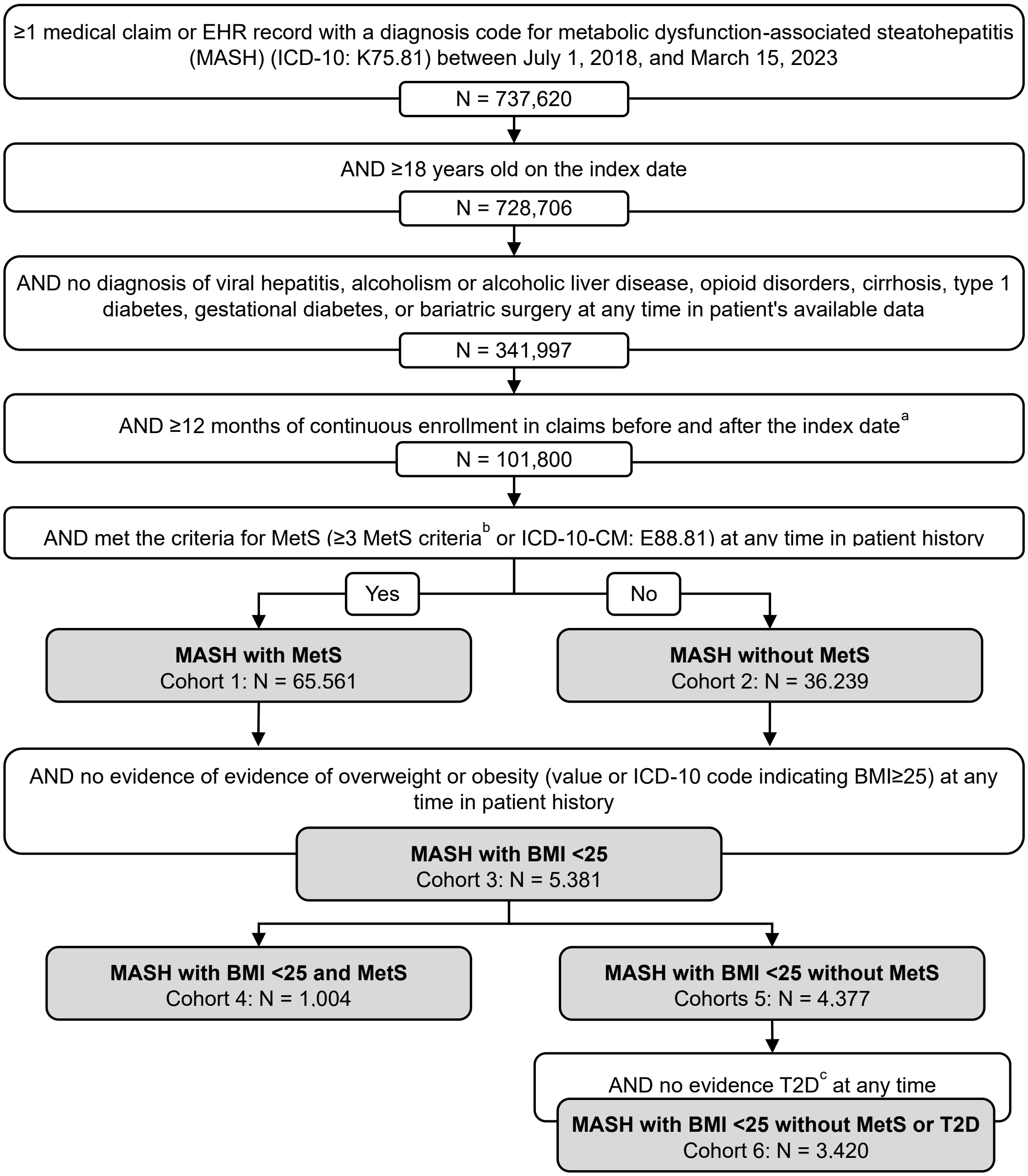

[a]The index date was the earliest MASH diagnosis during the patient selection period on which the individual met all study criteria. [b]Obesity, high blood pressure, hypercholesterolemia or hyperlipidemia, and type 2 diabetes as defined in the methods. [c]or elevated fasting glucose. BMI, body mass index; ICD-10-CM, International Classifications of Disease, Tenth Edition, Clinical Modification; MetS, metabolic syndrome; T2D, type 2 diabetes.

Supplementary Table 5. Demographic Characteristics – Sensitivity Analysis

	MASH with MetS	MASH without MetS	MASH with BMI <25	MASH with BMI <25 with MetS	MASH with BMI <25 without MetS	MASH with BMI <25 without MetS or T2D
	N = 65,561	N = 36,239	N = 5,381	N = 1,004	N = 4,377	N = 3,420
Age, mean (SD)	54.6 (12.7)	48.9 (14.3)	54.2 (14.7)	60.9 (12.5)	52.6 (14.7)	51.3 (14.9)
Female, n (%)	39,114 (59.7)	20,283 (56.0)	3,183 (59.2)	637 (63.4)	2,546 (58.2)	1,978 (57.8)
Race, n (%)						
Asian	3,070 (4.7)	2,213 (6.1)	635 (11.8)	161 (16.0)	474 (10.8)	346 (10.1)
African American/Black	2,940 (4.5)	1,090 (3.0)	109 (2.0)	16 (1.6)	93 (2.1)	62 (1.8)
Other	9,232 (14.1)	5,040 (13.9)	684 (12.7)	128 (12.7)	556 (12.7)	435 (12.7)
White	38,119 (58.1)	18,922 (52.2)	2,105 (39.1)	378 (37.6)	1,727 (39.5)	1,430 (41.8)
Unknown/Not Reported	12,200 (18.6)	8,974 (24.8)	1,848 (34.3)	321 (32.0)	1,527 (34.9)	1,147 (33.5)
Ethnicity, n (%)						
Hispanic	5,932 (9.0)	2,658 (7.3)	218 (4.1)	50 (5.0)	168 (3.8)	120 (3.5)
Non-Hispanic or Unknown	59,629 (91.0)	33,581 (92.7)	5,163 (95.9)	954 (95.0)	4,209 (96.2)	3,300 (96.5)
Geographic Region, n (%)						
Northeast	13,298 (20.3)	7,647 (21.1)	1,448 (26.9)	330 (32.9)	1,118 (25.5)	834 (24.4)
Midwest	11,555 (17.6)	6,655 (18.4)	742 (13.8)	98 (9.8)	644 (14.7)	523 (15.3)
South	27,299 (41.6)	13,687 (37.8)	1,761 (32.7)	327 (32.6)	1,434 (32.8)	1,161 (33.9)
West	13,393 (20.4)	8,246 (22.8)	1,430 (26.6)	249 (24.8)	1,181 (27.0)	902 (26.4)
Other/Unknown	16 (0.0)	4 (0.0)	0 (0.0)	0 (0.0)	0 (0.0)	0 (0.0)
Insurance Type, n (%)						
Commercial	39,134 (59.7)	24,634 (68.0)	3,425 (63.6)	524 (52.2)	2,901 (66.3)	2,364 (69.1)

	MASH with MetS	MASH without MetS	MASH with BMI <25	MASH with BMI <25 with MetS	MASH with BMI <25 without MetS	MASH with BMI <25 without MetS or T2D
	N = 65,561	N = 36,239	N = 5,381	N = 1,004	N = 4,377	N = 3,420
Medicare	14,651 (22.3)	4,723 (13.0)	1,158 (21.5)	351 (35.0)	807 (18.4)	551 (16.1)
Medicaid	11,701 (17.8)	6,820 (18.8)	789 (14.7)	128 (12.7)	661 (15.1)	499 (14.6)
Other/Unknown	75 (0.1)	62 (0.2)	9 (0.2)	1 (0.1)	8 (0.2)	6 (0.2)
Year of Index Date, n (%)						
2018	10,891 (16.6)	5,093 (14.1)	814 (15.1)	177 (17.6)	637 (14.6)	477 (13.9)
2019	14,419 (22.0)	7,456 (20.6)	1,129 (21.0)	224 (22.3)	905 (20.7)	688 (20.1)
2020	12,903 (19.7)	6,763 (18.7)	957 (17.8)	169 (16.8)	788 (18.0)	618 (18.1)
2021	14,959 (22.8)	9,079 (25.1)	1,300 (24.2)	211 (21.0)	1,089 (24.9)	866 (25.3)
2022	12,199 (18.6)	7,711 (21.3)	1,164 (21.6)	220 (21.9)	944 (21.6)	759 (22.2)
2023	190 (0.3)	137 (0.4)	17 (0.3)	3 (0.3)	14 (0.3)	12 (0.4)
Years of Follow-up, mean (SD)	2.8 (1.3)	2.7 (1.2)	2.7 (1.2)	2.8 (1.3)	2.7 (1.2)	2.6 (1.2)

MetS, metabolic syndrome; SD, standard deviation; T2D, type 2 diabetes

Supplementary Figure 4. Distribution of (A) Age and (B) Race[a] – Sensitivity Analysis.

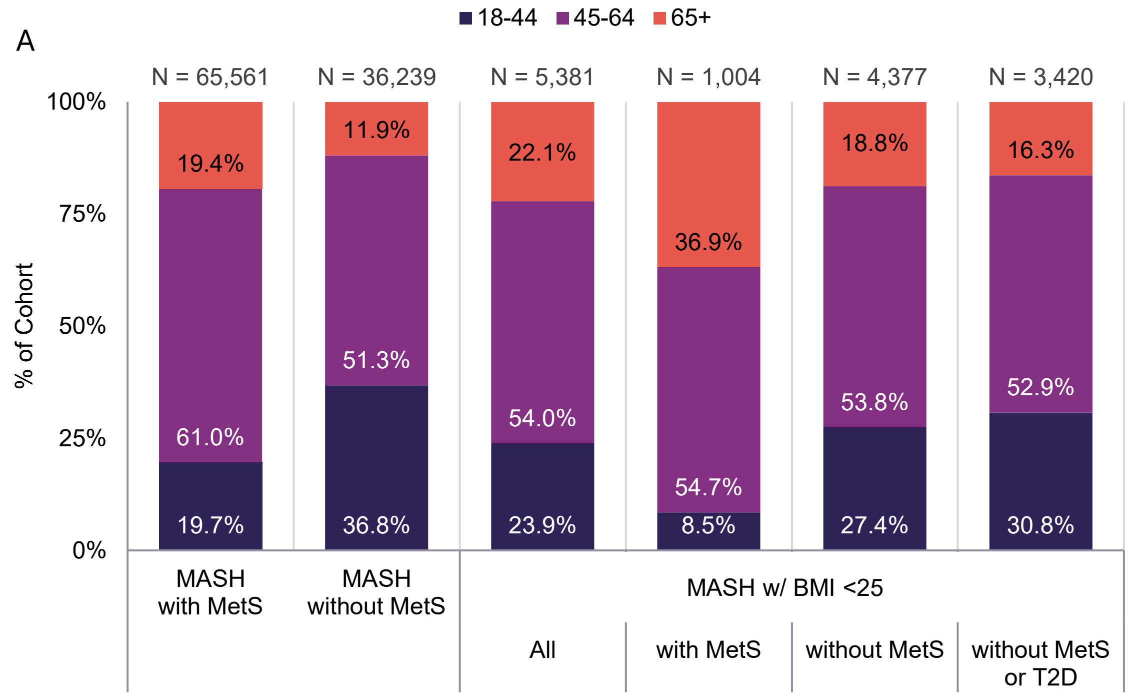

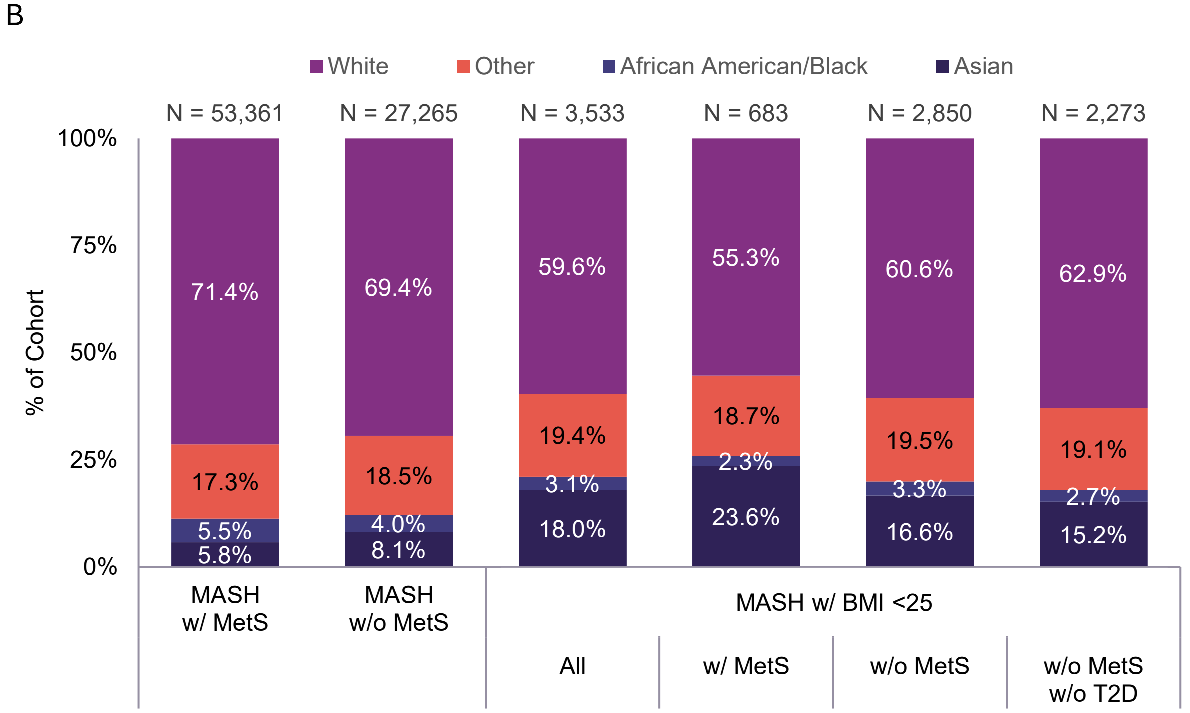

[a]Race was missing in 21,174 individuals and percentages are calculated only among those with available race data . MetS, metabolic syndrome; T2D, type 2 diabetes

Supplementary Table 6. Baseline Clinical Characteristics – Sensitivity Analysis

	MASH with MetS	MASH without MetS	MASH with BMI <25	MASH with BMI <25 with MetS	MASH with BMI <25 without MetS	MASH with BMI <25 without MetS or T2D
	N = 65,561	N = 36,239	N = 5,381	N = 1,004	N = 4,377	N = 3,420
Baseline BMI, n (%)	26,433 (40.3)	10,077 (27.8)	802 (14.9)	158 (15.7)	644 (14.7)	544 (15.9)
Baseline BMI, mean (SD)	34.0 (4.9)	30.9 (5.8)	21.9 (2.0)	22.2 (1.7)	21.8 (2.0)	21.7 (2.1)
MetS-related conditions, n (%)						
Hypertension (primary)	45,075 (68.8)	12,040 (33.2)	2,013 (37.4)	635 (63.2)	1,378 (31.5)	941 (27.5)
Hyperlipidemia	42,596 (65.0)	14,673 (40.5)	2,605 (48.4)	693 (69.0)	1,912 (43.7)	1,324 (38.7)
Obesity	38,986 (59.5)	12,209 (33.7)	0 (0.0)	0 (0.0)	0 (0.0)	0 (0.0)
Diabetes, type 2	32,343 (49.3)	2,479 (6.8)	1,096 (20.4)	553 (55.1)	543 (12.4)	0 (0.0)
Hypercholesterolemia	13,862 (21.1)	2,136 (5.9)	773 (14.4)	337 (33.6)	436 (10.0)	392 (11.5)
Metabolic syndrome[b]	3,223 (4.9)	0 (0.0)	64 (1.2)	64 (6.4)	0 (0.0)	0 (0.0)
Other Conditions, n (%)						
Thyroid disease	18,240 (27.8)	6,841 (18.9)	1,176 (21.9)	322 (32.1)	854 (19.5)	626 (18.3)
Anxiety	17,978 (27.4)	8,770 (24.2)	1,123 (20.9)	183 (18.2)	940 (21.5)	773 (22.6)
Sleep apnea	17,437 (26.6)	4,882 (13.5)	328 (6.1)	74 (7.4)	254 (5.8)	185 (5.4)
Depression	13,294 (20.3)	5,376 (14.8)	615 (11.4)	121 (12.1)	494 (11.3)	387 (11.3)
Chronic kidney disease	5,507 (8.4)	1,119 (3.1)	269 (5.0)	97 (9.7)	172 (3.9)	104 (3.0)
Anemia	4,187 (6.4)	1,600 (4.4)	303 (5.6)	85 (8.5)	218 (5.0)	164 (4.8)
Related Treatments, n (%)						
Any antihypertensive	44,488 (67.9)	12,844 (35.4)	2,056 (38.2)	605 (60.3)	1,451 (33.2)	985 (28.8)
GLP-1 RA	6,668 (10.2)	453 (1.3)	115 (2.1)	56 (5.6)	59 (1.3)	6 (0.2)

Other antidiabetic	27,702 (42.3)	2,551 (7.0)	813 (15.1)	414 (41.2)	399 (9.1)	21 (0.6)
Statin						
Low/moderate dose[c]	21,529 (32.8)	5,440 (15.0)	1,258 (23.4)	409 (40.7)	849 (19.4)	562 (16.4)
High dose[c]	10,263 (15.7)	2,012 (5.6)	412 (7.7)	131 (13.0)	281 (6.4)	174 (5.1)

BMI, body mass index; CCI, Charlson Comorbidity Index; GLP-1 RA, glucagon-like peptide-1 receptor agonists; MetS, metabolic syndrome; SD, standard deviation; T2D, type 2 diabetes. [a]Defined as patients with an ICD-10-CD diagnosis code of E88.81. [b]Statin dose intensity was calculated using peak average daily dose for any prescribed statin, with thresholds aligned with Quek, et al.[21] High doses were defined as follows: Atorvastatin ≥ 30mg daily; Rosuvastatin ≥ 15mg daily; Simvastatin ≥ 60mg daily.

Supplementary Table 7. Additional Baseline Clinical Characteristics – Sensitivity Analysis

	MASH with MetS	MASH without MetS	MASH with BMI <25	MASH with BMI <25 with MetS	MASH with BMI <25 without MetS	MASH with BMI <25 without MetS or T2D
	N = 65,561	N = 36,239	N = 5,381	N = 1,004	N = 4,377	N = 3,420
CCI Conditions, N (%)						
Mild liver disease[a]	32,087 (48.9)	16,109 (44.5)	2,083 (38.7)	415 (41.3)	1,668 (38.1)	1,329 (38.9)
Diabetes without chronic complication[a]	31,633 (48.2)	2,420 (6.7)	1,061 (19.7)	534 (53.2)	527 (12.0)	0 (0.0)
Chronic pulmonary disease	13,763 (21.0)	5,124 (14.1)	677 (12.6)	160 (15.9)	517 (11.8)	398 (11.6)
Diabetes with chronic complication[a]	10,563 (16.1)	567 (1.6)	308 (5.7)	172 (17.1)	136 (3.1)	1 (0.0)
Peripheral vascular disease	6,007 (9.2)	1,458 (4.0)	360 (6.7)	132 (13.1)	228 (5.2)	149 (4.4)
Renal disease[a]	5,349 (8.2)	1,072 (3.0)	262 (4.9)	92 (9.2)	170 (3.9)	103 (3.0)
Any malignancy (Incl. blood, excl. skin)	4,822 (7.4)	1,835 (5.1)	342 (6.4)	78 (7.8)	264 (6.0)	194 (5.7)
Cerebrovascular disease	3,851 (5.9)	1,042 (2.9)	244 (4.5)	78 (7.8)	166 (3.8)	115 (3.4)
Rheumatic disease	3,671 (5.6)	1,655 (4.6)	210 (3.9)	42 (4.2)	168 (3.8)	131 (3.8)
Congestive heart failure[a]	3,474 (5.3)	667 (1.8)	142 (2.6)	49 (4.9)	93 (2.1)	62 (1.8)
Myocardial infarction	1,659 (2.5)	295 (0.8)	69 (1.3)	20 (2.0)	49 (1.1)	29 (0.8)
Peptic ulcer disease	1,196 (1.8)	479 (1.3)	84 (1.6)	18 (1.8)	66 (1.5)	54 (1.6)
Metastatic solid tumor	665 (1.0)	340 (0.9)	56 (1.0)	12 (1.2)	44 (1.0)	30 (0.9)
Dementia	484 (0.7)	137 (0.4)	50 (0.9)	13 (1.3)	37 (0.8)	24 (0.7)
Hemiplegia or paraplegia	408 (0.6)	168 (0.5)	44 (0.8)	9 (0.9)	35 (0.8)	27 (0.8)
HIV/AIDS	278 (0.4)	167 (0.5)	36 (0.7)	7 (0.7)	29 (0.7)	24 (0.7)
Moderate or severe liver disease[a]	270 (0.4)	110 (0.3)	20 (0.4)	2 (0.2)	18 (0.4)	14 (0.4)

	MASH with MetS	MASH without MetS	MASH with BMI <25	MASH with BMI <25 with MetS	MASH with BMI <25 without MetS	MASH with BMI <25 without MetS or T2D
	N = 65,561	N = 36,239	N = 5,381	N = 1,004	N = 4,377	N = 3,420
Liver-Related Conditions (N, %)						
Autoimmune hepatitis	310 (0.5)	202 (0.6)	24 (0.4)	2 (0.2)	22 (0.5)	18 (0.5)
Gastroesophageal varices	96 (0.1)	45 (0.1)	8 (0.1)	0 (0.0)	8 (0.2)	6 (0.2)
Hepatic encephalopathy	45 (0.1)	20 (0.1)	4 (0.1)	1 (0.1)	3 (0.1)	2 (0.1)
Ascites	276 (0.4)	139 (0.4)	24 (0.4)	2 (0.2)	22 (0.5)	16 (0.5)
Cancers (N, %)						
Breast cancer	1,647 (2.5)	689 (1.9)	143 (2.7)	32 (3.2)	111 (2.5)	79 (2.3)
Prostate cancer	666 (1.0)	225 (0.6)	37 (0.7)	11 (1.1)	26 (0.6)	16 (0.5)
Colorectal cancer	409 (0.6)	196 (0.5)	39 (0.7)	9 (0.9)	30 (0.7)	23 (0.7)
Kidney cancer	302 (0.5)	80 (0.2)	6 (0.1)	1 (0.1)	5 (0.1)	4 (0.1)
Uterine cancer	285 (0.4)	87 (0.2)	15 (0.3)	2 (0.2)	13 (0.3)	12 (0.4)
Bladder cancer	205 (0.3)	46 (0.1)	11 (0.2)	4 (0.4)	7 (0.2)	3 (0.1)
Ovarian cancer	119 (0.2)	65 (0.2)	12 (0.2)	1 (0.1)	11 (0.3)	10 (0.3)
Liver cancer	90 (0.1)	25 (0.1)	4 (0.1)	1 (0.1)	3 (0.1)	2 (0.1)
Hepatocellular carcinoma	37 (0.1)	11 (0.0)	2 (0.0)	1 (0.1)	1 (0.0)	0 (0.0)
Pancreatic cancer	78 (0.1)	39 (0.1)	12 (0.2)	2 (0.2)	10 (0.2)	8 (0.2)
Stomach cancer	34 (0.1)	10 (0.0)	1 (0.0)	0 (0.0)	1 (0.0)	1 (0.0)

MetS, metabolic syndrome; SD, standard deviation; T2D, type 2 diabetes a) The code sets to define these conditions overlap with those used to define the study cohorts.

Supplementary Figure 5. Annualized Mean (Standard Deviation) All-cause Healthcare Costs in the Variable Length Follow-up Period – Sensitivity Analysis.

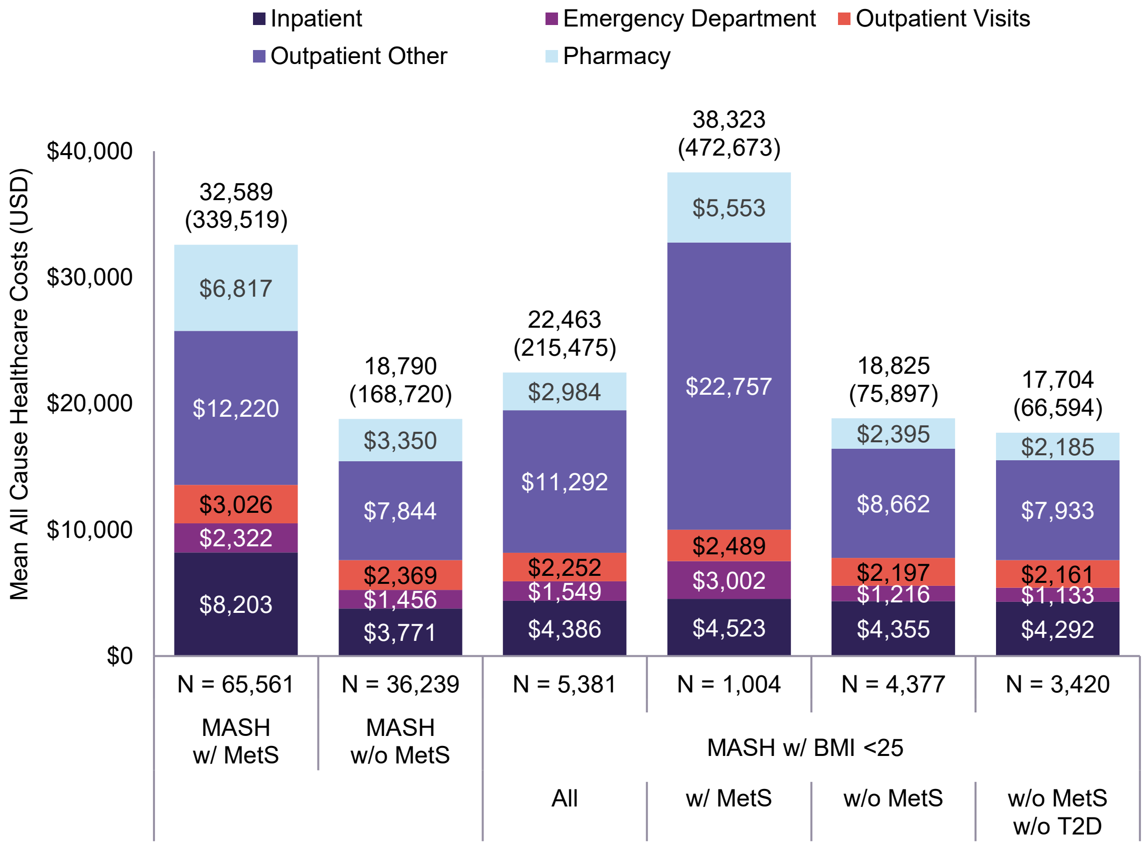

MetS, metabolic syndrome; T2D, type 2 diabetes

Supplementary Table 8. All-Cause Healthcare Resource Utilization in the Variable Follow-Up Period Reported as Per-Person Per-Year – Sensitivity Analysis

	MASH with MetS	Mash without MetS	MASH with BMI <25	MASH with BMI <25 with MetS	MASH with BMI <25 without MetS	MASH with BMI <25 without MetS or T2D
	N = 65,561	N = 36,239	N = 5,381	N = 1,004	N = 4,377	N = 3,420
Inpatient admission						
Had an admission, n (%)	15,436 (23.5)	5,395 (14.9)	811 (15.1)	191 (19.0)	620 (14.2)	452 (13.2)
Number of admissions, mean (SD)	0.3 (1.2)	0.1 (0.9)	0.2 (1.6)	0.4 (2.6)	0.2 (1.3)	0.2 (1.1)
Length of stay per hospitalized patient, mean (SD)	7.9 (21.8)	7.2 (21.5)	9.8 (35.5)	11.0 (31.2)	9.4 (36.8)	7.1 (20.9)
Costs, mean (SD) among all patients	$8,203 ($206,151)	$3,771 ($50,242)	$4,386 ($40,185)	$4,523 ($22,839)	$4,355 ($43,192)	$4,292 ($46,822)
Costs, median among all patients	$0	$0	$0	$0	$0	$0
Costs, mean (SD) for patients with an admission	$34,237 ($420,088)	$24,626 ($126,378)	$28,300 ($98,703)	$22,935 ($47,145)	$29,969 ($109,870)	$31,432 ($123,295)
Costs, median for patients with an admission	$7,764	$6,585	$7,710	$8,336	$7,229	$6,757
Emergency Department (ED)						
Had an ED visit, n (%)	32,131 (49.0)	14,593 (40.3)	1,807 (33.6)	363 (36.2)	1,444 (33.0)	1,111 (32.5)
Number of ED visits, mean (SD) among all patients	0.6 (1.5)	0.4 (1.0)	0.3 (0.8)	0.4 (0.8)	0.3 (0.8)	0.3 (0.8)
Costs, mean (SD) among all patients	$2,322 ($13,896)	$1,456 ($7,044)	$1,549 ($16,455)	$3,002 ($33,326)	$1,216 ($8,805)	$1,133 ($4,686)
Costs, median among all patients	$0	$0	$0	$0	$0	$0
Costs, mean (SD) for patients with an ED visit	$4,744 ($19,570)	$3,621 ($10,752)	$4,618 ($28,161)	$8,302 ($55,026)	$3,691 ($15,040)	$3,495 ($7,712)
Costs, median for patients with an ED visit	$1,455	$1,219	$1,147	$1,473	$1,063	$1,116

	MASH with MetS	Mash without MetS	MASH with BMI <25	MASH with BMI <25 with MetS	MASH with BMI <25 without MetS	MASH with BMI <25 without MetS or T2D
	N = 65,561	N = 36,239	N = 5,381	N = 1,004	N = 4,377	N = 3,420
Outpatient (OP) Visits						
Had an OP visit, n (%)	65,422 (99.8)	36,121 (99.7)	5,346 (99.3)	996 (99.2)	4,350 (99.4)	3,400 (99.4)
Number of OP visits, mean (SD)	16.8 (13.6)	12.9 (11.8)	12.5 (12.1)	14.2 (15.2)	12.1 (11.2)	12.0 (11.4)
Costs, mean (SD) among all patients	$3,026 ($8,401)	$2,369 ($7,065)	$2,252 ($5,093)	$2,489 ($4,345)	$2,197 ($5,249)	$2,161 ($5,096)
Costs, median among all patients	$1,443	$1,011	$999	$1,224	$946	$935
OP Other						
Had an OP other service, n (%)	65,347 (99.7)	35,952 (99.2)	5,334 (99.1)	1,001 (99.7)	4,333 (99.0)	3,383 (98.9)
Number of OP other services, mean (SD)	15.6 (26.8)	10.2 (17.3)	11.2 (23.2)	15.4 (34.4)	10.2 (19.7)	9.7 (17.9)
Costs, mean (SD) among all patients	$12,220 ($94,793)	$7,844 ($40,477)	$11,292 ($208,065)	$22,757 ($467,133)	$8,662 ($55,950)	$7,933 ($40,427)
Costs, median among all patients	$2,934	$1,786	$1,721	$2,286	$1,612	$1,571
Pharmacy						
Had a pharmacy claim, n (%)	64,880 (99.0)	35,435 (97.8)	5,216 (96.9)	990 (98.6)	4,226 (96.6)	3,299 (96.5)
Number of unique NDCs, mean (SD)	13.2 (9.1)	8.3 (6.6)	8.1 (6.5)	10.4 (7.0)	7.6 (6.2)	7.1 (5.8)
Costs, mean (SD) among all patients	$6,817 ($247,893)	$3,350 ($153,539)	$2,984 ($25,165)	$5,553 ($54,939)	$2,395 ($9,183)	$2,185 ($9,480)
Costs, median among all patients	$1,223	$302	$321	$605	$278	$228

	MASH with MetS	Mash without MetS	MASH with BMI <25	MASH with BMI <25 with MetS	MASH with BMI <25 without MetS	MASH with BMI <25 without MetS or T2D
	N = 65,561	N = 36,239	N = 5,381	N = 1,004	N = 4,377	N = 3,420
Total Costs Among All Patients						
Total Costs, mean (SD)	$32,589 ($339,519)	$18,790 ($168,720)	$22,463 ($215,475)	$38,323 ($472,673)	$18,825 ($75,897)	$17,704 ($66,594)
Total Costs, median (IQR)	$11,300 ($4,436-$27,074)	$5,689 ($2,126-$15,520)	$5,589 ($2,135-$15,047)	$8,275 ($3,178-$20,292)	$5,094 ($1,962-$13,867)	$4,758 ($1,840-$13,038)
Total Costs After Removing Top 1% of Spenders						
Total Costs, mean (SD)	$21,785 ($30,708)	$14,402 ($25,172)	$14,239 ($25,661)	$17,911 ($28,244)	$13,401 ($24,959)	$13,007 ($24,635)
Total Costs, median (IQR)	$10,960 ($4,338-$25,757)	$5,594 ($2,100-$15,025)	$5,453 ($2,099-$14,408)	$7,963 ($3,091-$19,729)	$4,963 ($1,928-$13,187)	$4,614 ($1,818-$12,693)
